# Highly Efficient Exciton Modulation in MoSe_2_/PdSe_2_ Heterostructures

**DOI:** 10.1021/acs.nanolett.6c02581

**Published:** 2026-09-23

**Authors:** Petr Rozhin, Muhammad Sufyan Ramzan, Emma Contin, Danae Katrisioti, Till Weickhardt, Micol Bertolotti, Nouha Loudhaief, Bing Wu, Zdeněk Sofer, Takashi Taniguchi, Kenji Watanabe, Leonardo Puppulin, Caterina Cocchi, Ioannis Paradisanos, Giancarlo Soavi, Stefano Dal Conte, Giovanni Antonio Salvatore, Domenico De Fazio

**Affiliations:** † Department of Molecular Sciences and Nanosystems, 19047 Ca’ Foscari University of Venice, 30172 Venice, Italy; ‡ Institute of Condensed Matter Theory and Optics, 11233 Friedrich Schiller University Jena, 07743 Jena, Germany; § Department of Materials Science and Engineering, University of Crete, 70013 Heraklion, Crete, Greece; ∥ Institute of Electronic Structure and Laser, 124215 Foundation for Research and Technology—Hellas, 70013 Heraklion, Crete, Greece; ⊥ Institute of Solid State Physics, 9378 Friedrich Schiller University Jena, 07743 Jena, Germany; # Department of Physics, 18981 Politecnico di Milano, I-20133 Milano, Italy; ∇ Department of Inorganic Chemistry, 9318 University of Chemistry and Technology Prague, 166 28 Prague 6, Czech Republic; ○ Research Center for Materials Nanoarchitectonics, 52735 National Institute for Materials Science, 1-1 Namiki, Tsukuba 305-0044, Japan; ◆ Research Center for Electronic and Optical Materials, National Institute for Materials Science, 1-1 Namiki, Tsukuba 305-0044, Japan

**Keywords:** MoSe_2_, PdSe_2_, heterostructure, exciton, photoluminescence

## Abstract

We report a pronounced ∼6-fold enhancement of room-temperature A-exciton emission in a type-I MoSe_2_/PdSe_2_ van der Waals heterostructure, yielding a photoluminescence quantum yield of 6%, as compared to ∼1% for as-exfoliated monolayer MoSe_2_. This unusual enhancement is accompanied by strong quenching of the B exciton, consistent with interlayer electronic coupling that redistributes exciton populations toward the radiative A-exciton channel. Power- and temperature-dependent measurements reveal a suppression of exciton–exciton annihilation and a crossover to quenched emission at a low temperature, indicating a redistribution of exciton relaxation pathways. Photoluminescence excitation spectroscopy further reveals a broadband enhancement spanning 450–725 nm. These results demonstrate that interlayer electronic coupling can be used as an efficient means to redirect exciton populations toward radiative channels, enhancing emission efficiency in two-dimensional semiconductors without chemical modification or significant strain.

## Graphical abstract

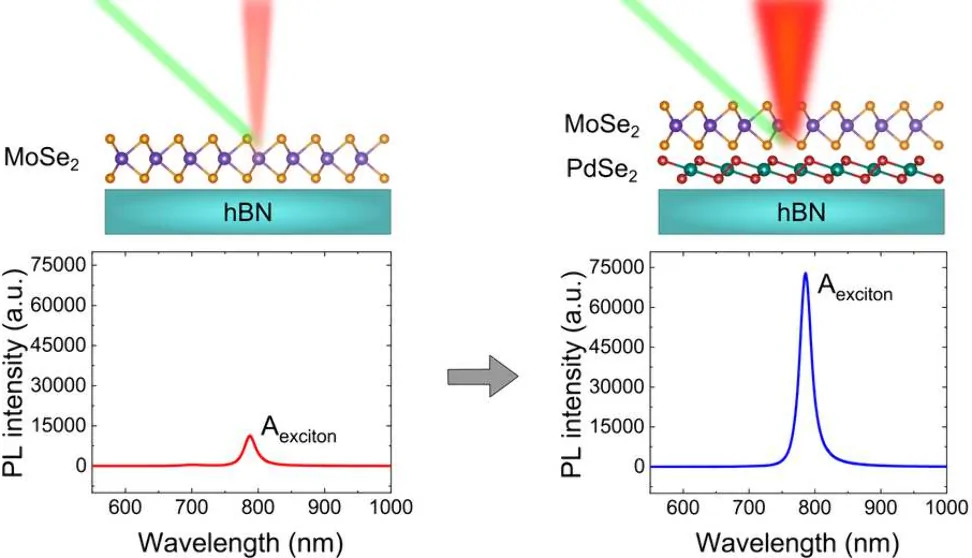

Strong, stable light emission is essential for optoelectronic devices, such as light-emitting diodes (LEDs),^1^ lasers,^2^ and single-photon sources.^3^ Efficient emission occurs when excitons recombine radiatively, consistently producing photons, rather than being lost to heat,^4^ defect trapping,^5^ phonon-assisted decay,^6^ and Auger recombination.^7,8^ Suppression of these non-radiative pathways increases the photoluminescence quantum yield (PLQY), defined as the ratio of emitted photons to absorbed photons, thereby improving the efficiency of light-emitting devices and reducing their power consumption, both key aspects in modern photonic components.^9–12^

Transition metal dichalcogenides (TMDs), such as MoS_2_, MoSe_2_, WS_2_, and WSe_2_, have emerged as promising platforms for next-generation optoelectronics owing to their unique properties, including a direct bandgap in the monolayer limit,^13^ strong photoluminescence (PL),^14^ large exciton binding energies (typically ∼300–600 meV),^15,16^ pronounced spin–orbit coupling,^17^ mechanical flexibility,^18^ and the ability to form van der Waals (vdW) heterostructures with novel functionalities.^19,20^ Furthermore, their atomic thickness makes their optical response highly sensitive to the surrounding dielectric environment.^21,22^ While this sensitivity enables powerful control of excitonic properties through dielectric engineering, it also makes emission strongly dependent on defects,^23^ substrate disorder,^24^ and interface quality.^25^

Several approaches have been employed to enhance the PLQY of monolayer TMDs, including encapsulation,^25–28^ chemical passivation,^23,29,30^ and strain engineering.^31,32^ Encapsulation with dodecanol passivates Se vacancies and suppresses substrate-induced non-radiative recombination, enabling PLQY values from 6.2% up to ∼22% at 5 K in MoSe_2_.^28^ In addition, the thickness of the hexagonal boron nitride (hBN) encapsulation layer can control the exciton radiative rate via the Purcell effect (where the local optical environment alters the spontaneous emission rate of excitons).^33^ Chemical treatments, such as bis­(trifluoromethane) sulfonimide (TFSI) passivation, have also been used to increase the PLQY of MoS_2_ from ∼0.8% to over 95% by reducing defect-mediated recombination.^23^ Strain engineering exploits spatially graded strain fields to funnel excitons toward regions of minimum bandgap, with reported PL intensity increasing by a factor of ∼2 in MoS_2_ and ∼2.8 in WSe_2_.^31,32^ However, these approaches often rely on chemical treatments that degrade rapidly under ambient exposure^29,30^ or on uniform tensile strain that drives a direct-to-indirect bandgap transition, thereby suppressing PL.^34^ Additionally, achieving high PL via hBN encapsulation typically requires vacuum annealing at ∼1000 °C,^27^ a temperature incompatible with the use of pre-patterned electrodes, polymer substrates, and post-integration device architectures. These limitations motivate the need for PLQY control through non-invasive interlayer engineering.

Forming vdW stacking enables tuning of electronic and optical properties through interlayer electronic coupling^20^ rather than solely through dielectric screening as in encapsulation.^35^ In type-I heterostructures, both the conduction band minimum (CBM) and valence band maximum (VBM) of one layer lie within the gap of the other layer, confining electrons and holes to the same material and enhancing radiative recombination.^36^ In contrast, type-II heterostructures exhibit staggered band alignment, where the CBM and VBM reside in different layers, spatially separating electrons and holes and typically reducing the PL intensity. The PL enhancement in type-I heterostructures is typically attributed to exciton funneling, whereby carriers generated in the wider bandgap layer transfer into the narrower bandgap emissive layer and accumulate there.^36,37^ This mechanism has been widely reported in systems combining an emissive TMD, such as MoSe_2_ or WS_2_, with a non- or weakly emissive material, for example, in FePS_3_/MoSe_2_ (showing a ∼20-fold enhancement of room-temperature PL emission from monolayer MoSe_2_)^38^ and PbI_2_/WS_2_ (exhibiting a ∼2-fold PL enhancement relative to bare monolayer WS_2_)^39^ heterostructures.

Interestingly, an unusual scenario has been demonstrated in a type-I heterostructure in which a strongly absorbing, non-luminescent narrow-gap SnSe_2_ multilayer underlies a wider-gap monolayer MoS_2_.^41^ In that system, the MoS_2_ PL is enhanced by up to 14-fold via a mechanism named Förster resonance energy transfer (FRET), that is, non-radiative transfer of the energy of photoexcited electron–hole pairs in SnSe_2_ to the MoS_2_ monolayer through near-field dipole–dipole coupling.^41^ In type-I band alignments, one would expect carrier funneling into the narrower gap layer, which should quench emission from the wider bandgap TMD by depleting its photoexcited carriers. In this sense, MoS_2_/SnSe_2_ represents an unusual situation, as a strong interlayer energy transfer outweighs the PL loss pathways associated with charge transfer. This result established a general design principle: pairing a strongly absorbing, weakly emissive narrow-gap layer (SnSe_2_) with a highly luminescent wider gap TMD monolayer (MoS_2_) can enhance rather than quench emission from the TMD. However, FRET efficiency in that system relies critically on the near resonance between the direct transition of SnSe_2_ and the excitonic gap of MoS_2_, a condition that is not easily generalizable to other material combinations.

Here, we fabricate a vdW heterostructure combining a narrow-gap PdSe_2_ multilayer with a monolayer of MoSe_2_. PdSe_2_ exhibits strong broadband absorption but weak PL.^42,43^ Unlike SnSe_2_, PdSe_2_ does not exhibit a strong spectral resonance with the dominant excitonic transitions of MoSe_2_, placing the system in a regime governed by interlayer electronic hybridization rather than dipole–dipole energy transfer. Its narrow-gap electronic structure and strong polarizability enable interactions beyond dielectric screening, in contrast to hBN or SiO_2_. MoSe_2_ is selected for its strong room-temperature emission^44^ and well-resolved A and B excitons (∼200 meV splitting),^45,46^ enabling sensitive tracking of exciton relaxation pathways.^47^ We investigate how interlayer coupling in a MoSe_2_/PdSe_2_/hBN vdW heterostructure modifies exciton relaxation and PLQY. We observe a pronounced enhancement of the MoSe_2_ A-exciton PL, with the extracted PLQY increasing from ∼1% to 6%.

Our approach exploits interlayer coupling in a vdW heterostructure, preserving the intrinsic material properties. In addition to enhanced A-exciton emission, the heterostructure exhibits strong B-exciton quenching, which we attribute to interlayer hybridization of MoSe_2_ with PdSe_2_ due to the enabling of additional relaxation pathways. As a result, exciton populations are redistributed toward the lower energy A exciton, where radiative recombination is favored, leading to an enhanced A-exciton emission. These findings demonstrate that PdSe_2_ can serve as an effective platform for engineering exciton dynamics and enhancing the PL efficiency in two-dimensional semiconductors without significant strain or chemical functionalization. Enhanced A-exciton PL in MoSe_2_/PdSe_2_ arises from interlayer coupling that requires near-resonant valence-band alignment and low substrate density of states at the A-exciton energy, ensuring efficient radiative recombination.

We begin our analysis with the inspection of the optical image of a MoSe_2_/PdSe_2_/hBN heterostructure, which is shown in Figure 1a. The individual hBN, PdSe_2_, and MoSe_2_ flakes, as well as the overlapping MoSe_2_/PdSe_2_/hBN heterostructure area, are outlined by yellow dashed lines, light blue dashed lines, red dashed lines, and overlapping polygons, respectively. The thickness and layer quality of PdSe_2_ and MoSe_2_ were characterized by Raman spectroscopy and atomic force microscopy (AFM) in Figures S1 and S2, respectively. The sample consists of a monolayer trigonal prismatic phase MoSe_2_ flake partially overlapping a hexalayer (6L) PdSe_2_ flake on a hBN substrate. At this thickness, PdSe_2_ retains a finite bandgap of ∼1.0 eV while exhibiting strong broadband absorption that increases with the layer number.^48^ In contrast, monolayer PdSe_2_ has a larger bandgap (∼1.3 eV)^49,50^ and a weaker band offset with MoSe_2_,^51,52^ limiting interlayer electronic coupling. On the other hand, thicker PdSe_2_ flakes approach semimetallic behavior (bulk bandgap of ∼0.03 eV)^49^ and would possess a higher density of states, which, in turn, could promote non-radiative recombination.^53^ In the following, we distinguish between two regions of the same MoSe_2_ monolayer: (i) the heterostructure (HS) region, where MoSe_2_ lies on PdSe_2_, and (ii) the reference monolayer (1L) region, where MoSe_2_ lies directly on hBN without PdSe_2_ underneath. Both regions, therefore, contain 1L MoSe_2_ but differ in the presence or absence of the PdSe_2_ layer.

**Figure 1:**
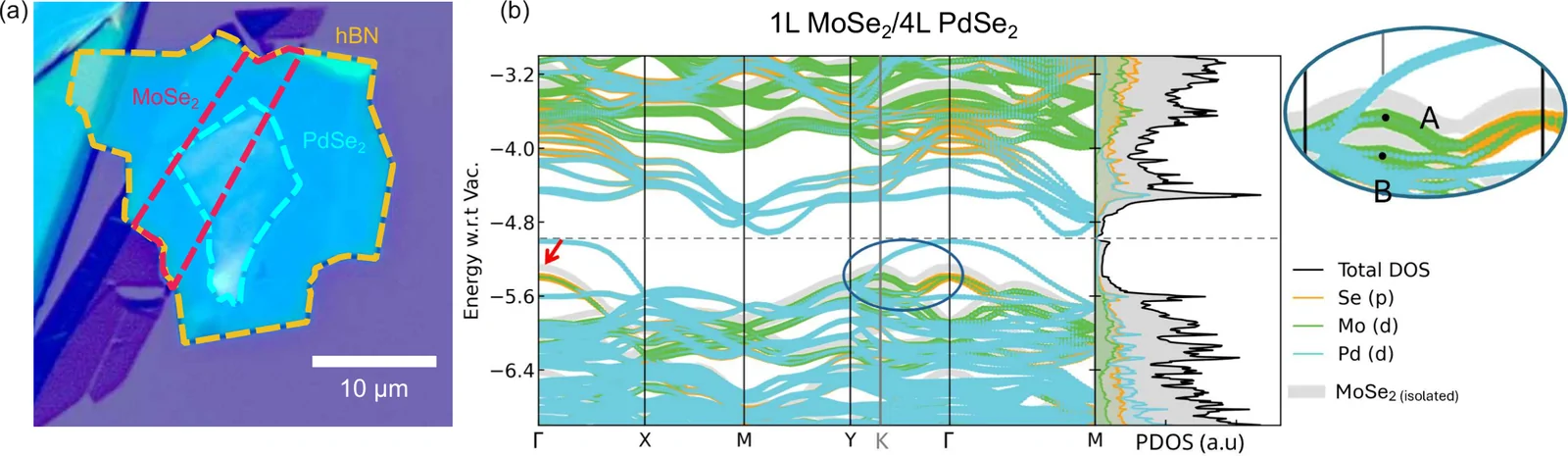
Structural and electronic properties of the MoSe_2_/PdSe_2_ heterostructure on a hBN substrate: (a) Optical image of the MoSe_2_/PdSe_2_/hBN heterostructure and (b) electronic band structures and projected density of states (PDOS) of a model heterostructure formed by 1L MoSe_2_ and 4L PdSe_2_. The vacuum level is set to 0 eV, and the Fermi level is marked with a horizontal dashed line in the mid-gap. The red arrow indicates the valence edges of 1L MoSe_2_. The vertical bar between the high-symmetry points Y and Γ indicates the high-symmetry point K of the unfolded primitive hexagonal lattice.^40^ The inset shows the zoomed-in region with spin-split A- and B-exciton valleys of MoSe_2_.

To clarify the nature of interlayer electronic interactions in this system, we performed *ab initio* simulations on a model system, including 1L MoSe_2_ stacked on tetralayer (4L) PdSe_2_ (Figure S3). The 4L PdSe_2_ model was adopted as a computationally efficient approximation of the experimentally investigated 6L PdSe_2_ because of their similar electronic structures (Figure S4). In particular, the band-edge positions and work functions differ by less than ∼0.1 eV (Table S1), indicating that the interlayer hybridization mechanism is not expected to be significantly affected by the reduced PdSe_2_ thickness. The electronic structure of the 1L MoSe_2_/4L PdSe_2_ interface exhibits an almost vanishing indirect bandgap, with both VBM and CBM dominated by PdSe_2_ states (Figure 1b). The highest occupied states with a MoSe_2_ character are buried in the valence band of PdSe_2_, between the VBM and the VBM–1 (the energy state located directly below the VBM) at the Γ point. The interaction with the underlying PdSe_2_ layer downshifts the uppermost valence bands of MoSe_2_ by about 100 meV relative to the isolated 1L. Moreover, close to the K valley of pristine MoSe_2_ (inset of Figure 1b), where the A and B excitons originate, there are signatures of strong hybridization with PdSe_2_ bands. The strength of this interlayer electronic hybridization and the magnitude of the density of states are discussed in the “Quantifying interlayer hybridization” subsection of the Supporting Information (see Figures S5 and S6). This hybridization generates mixed electronic states that are expected to quench excitonic resonances in MoSe_2_. Beyond opening new optical transition channels, this state mixing is anticipated to be particularly pronounced for the B exciton, stemming from an even deeper valley interacting with the PdSe_2_ valence region. Such interlayer coupling modifies the relaxation pathways of photoexcited carriers, enabling additional non-radiative interlayer relaxation channels, which compete against the intrinsic B → A intralayer exciton relaxation. A schematic illustration of the proposed carrier relaxation pathways, differentiating electron and hole dynamics under both resonant and non-resonant excitation, is provided in Figure S7. This alters exciton population dynamics, leading to a redistribution of carriers between A-exciton emission and interlayer decay channels, which may contribute to the enhanced A-exciton emission highlighted below.

To validate this interpretation, we performed control calculations on an analogous 1L MoS_2_/4L PdSe_2_ HS (Figure S8a). In this case, the valence states of MoS_2_ are energetically deeper and do not hybridize as strongly with the valence bands of PdSe_2_. Consequently, exciton quenching is less pronounced, consistent with equivalent control measurements (Figure S8b and c).

To investigate how band alignment affects excitonic emission, we performed PL measurements of the HS region. Figure 2a shows a pronounced enhancement of the A-exciton PL in the HS region. Under 514 nm excitation, the A-exciton PL intensity increases by a factor ∼6× relative to the 1L region. Assuming comparable optical absorption at the excitation wavelength and identical light extraction efficiencies, we estimate an external PLQY of approximately 6% for the HS, using the reported internal PLQY of 1% for as-exfoliated 1L MoSe_2_ as a benchmark.^54^ Notably, while the absolute PL intensity of the 1L is higher under 633 nm excitation due to its proximity to the A-exciton resonance, the relative enhancement factor decreases to ∼4× (Figure S9). This suggests that the HS-induced enhancement is more pronounced under off-resonant excitation conditions.

**Figure 2:**
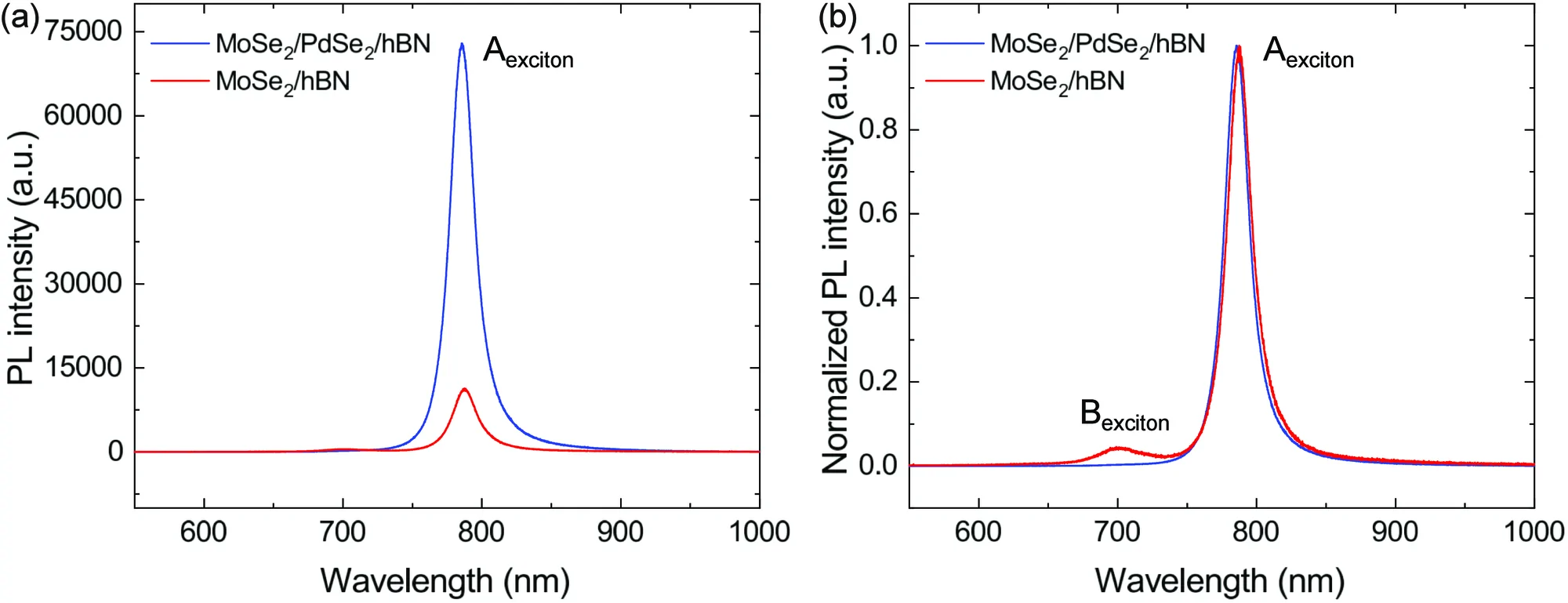
PL spectra of the 1L region and the HS region: (a) A-exciton PL enhancement in the HS region at 514 nm excitation and (b) B-exciton quenching in the HS region. PL intensity is reported in arbitrary units (a.u.), corresponding to detector counts.

In type-I TMD HSs, charge carriers transfer from the wider bandgap layer to the narrower bandgap emissive layer, resulting in enhanced radiative recombination.^55,56^ Although DFT calculations indicate a type-I band alignment for the MoSe_2_/PdSe_2_ HS (Figure 1b), the observed PL enhancement occurs in wider bandgap MoSe_2_. This contradicts the expected carrier funneling mechanism, as injection into narrower gap PdSe_2_ would deplete the MoSe_2_ exciton population and suppress emission. Similarly, while dielectric screening from a dielectric substrate like hBN can influence the excitonic behavior of TMDs, it fails to account for the PL enhancement observed in this HS. The specificity of this effect is underscored by the fact that PdSe_2_ does not enhance PL in MoS_2_ (Figure S8b and c), ruling out generic substrate-induced passivation. Instead, we attribute the enhancement to specific electronic interactions between PdSe_2_ and MoSe_2_. With support for this interpretation, Figure 2b reveals a strong quenching of the B-exciton emission in the HS region, consistent with the interlayer hybridization predicted by our DFT calculations. Furthermore, the minimal blue shift (∼3 meV)^57^ and the absence of a clear trion (charged exciton) enhancement^58^ suggest that charge-transfer-induced doping is negligible. Consequently, neither simple carrier funneling nor doping variations can be considered the dominant drivers of the observed PL intensity modification.

To prove the robustness of our analysis, we examined the consistency of this behavior across the HS. As shown in Figure 3a, the PL emission is uniform across the entire HS region, demonstrating the spatiotemporal spectral homogeneity of the A-exciton PL. The PL mapping in Figure 3b further confirms low B-exciton population throughout the HS area.

**Figure 3:**
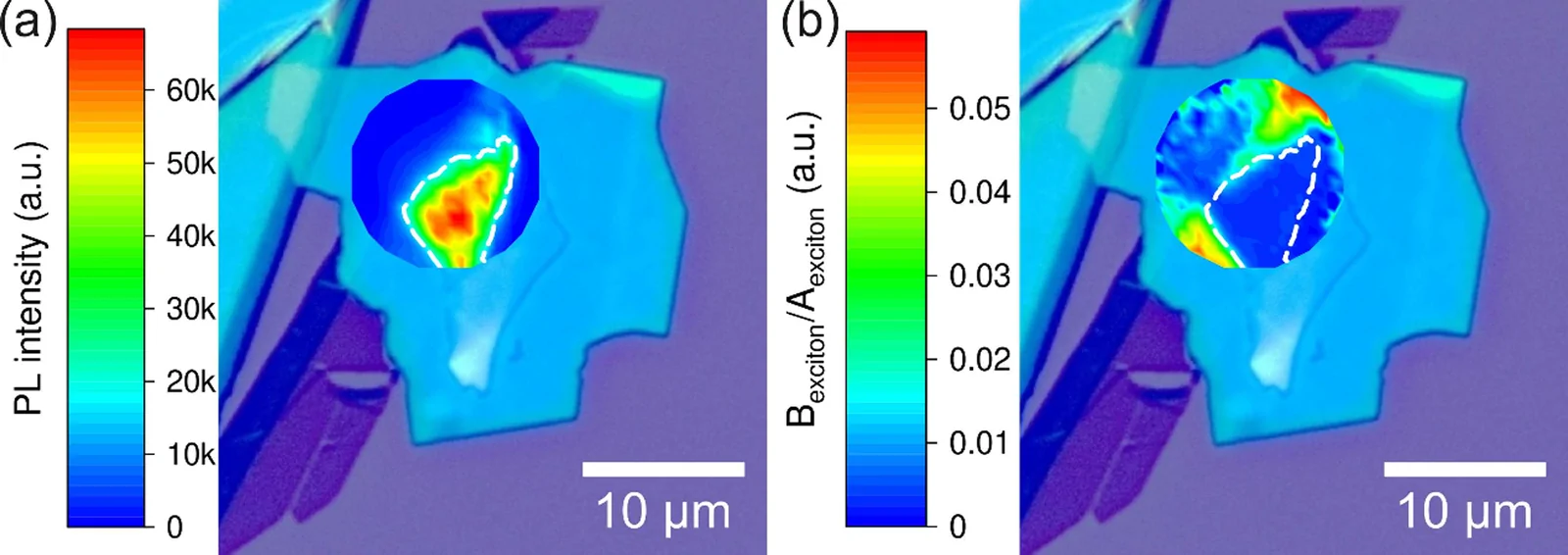
MoSe_2_/PdSe_2_ HS PL mapping: (a) A-exciton PL intensity map, *I*_A_, and (b) B/A-exciton intensity ratio map, *I*_B_/*I*_A_, where the HS area is highlighted with a dashed white line.

To further probe the underlying mechanisms of the observed A-exciton enhancement and B-exciton quenching, we investigated the dependence of the PL intensity on excitation power and temperature. Because 1L MoSe_2_ forms the top layer of the stack (MoSe_2_/PdSe_2_/hBN), the incident laser power is absorbed directly by MoSe_2_ without prior attenuation in both regions. Furthermore, the absence of trion emission and the minimal spectral shift of ∼3 meV confirm that effective carrier densities remain comparable between the two regions. The data presented in Figure 4 are well-described by a power-law dependence (*I* ∝ *P* ^α^). As shown in Figure 4a, the PL intensity of both the 1L region and the HS region increases sublinearly with excitation power, yielding exponents of α ≃ 0.599 and 0.738, respectively. An exponent significantly smaller than unity (α < 1) in the 1L region typically indicates that relaxation dynamics are dominated by exciton–exciton annihilation (EEA), a process whereby two excitons interact and one or both recombine non-radiatively, reducing the PL intensity at high excitation densities.^59^ The increase of the exponent to α ≃ 0.738 in the HS region suggests a suppression of EEA, consistent with interlayer hybridization modifying exciton relaxation pathways.

**Figure 4:**
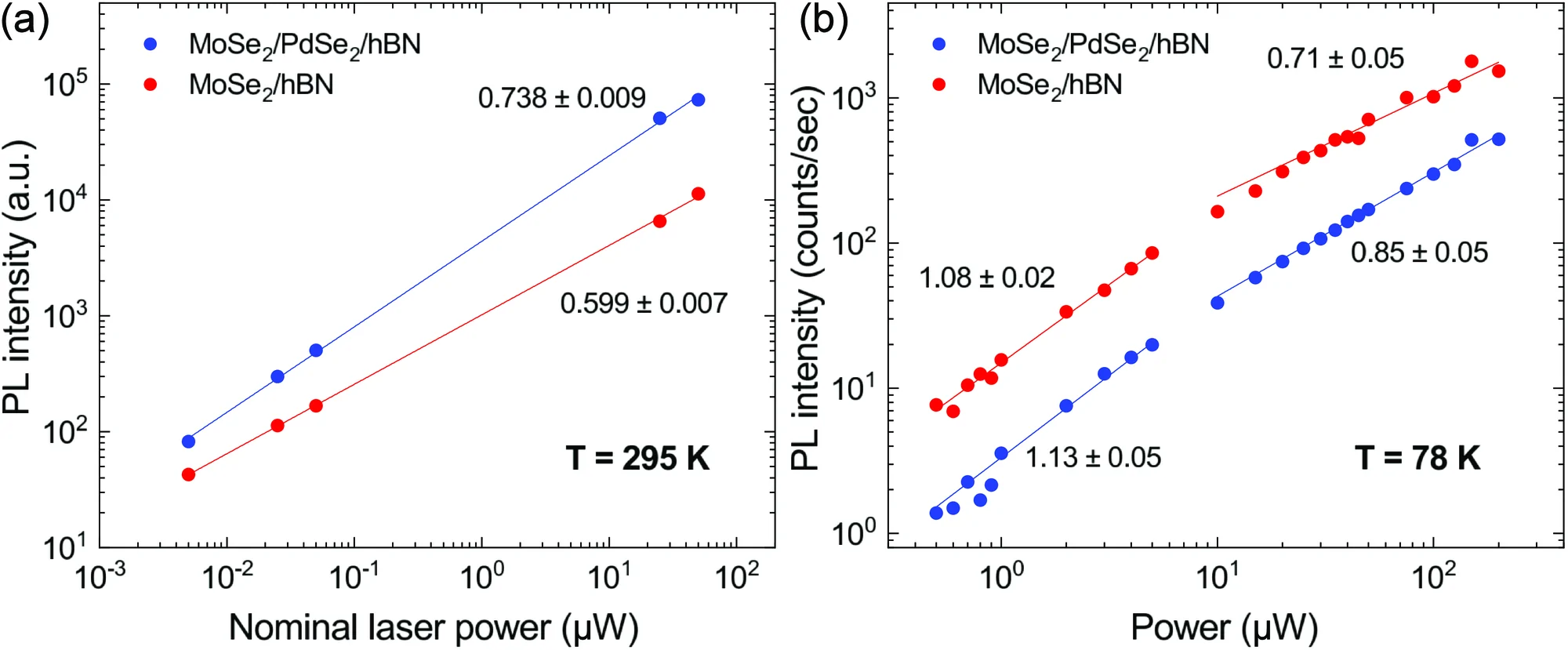
Laser power-dependent PL spectra of the A exciton at (a) room temperature and (b) cryogenic temperature. The reported power denotes the incident laser power at the sample.

At cryogenic temperatures, Figure 4b reveals two distinct regimes. In the low-power region, the PL intensity scales nearly linearly with excitation power, yielding exponents of α ≃ 1.08 for the 1L region and α ≃ 1.13 for the HS region. At higher excitation powers, the response becomes sublinear, with α ≃ 0.71 and 0.85, respectively, which is attributed to EEA at high carrier densities.^60^ Importantly, unlike the room-temperature behavior, the large PL enhancement disappears at 78 K, with the HS region exhibiting lower intensity than the 1L region. This observation indicates that the mechanisms responsible for the A-exciton enhancement are strongly temperature-dependent, consistent with a temperature-dependent redistribution of exciton relaxation dynamics that favors radiative recombination at intermediate temperatures (∼200–300 K) and weakens at low temperatures (<200 K).

Figure 5 presents temperature-dependent PL measurements of the HS region and the 1L region. The 1L region exhibits a conventional thermal quenching behavior,^44^ with the PL intensity decreasing as the temperature increases (Figure 5a). This behavior is attributed to the activation of non-radiative recombination processes,^44^ as exciton–phonon interactions at elevated temperatures provide sufficient thermal energy for excitons to access non-radiative pathways. Concurrently, the PL peak energy red shifts with an increasing temperature, consistent with lattice thermal expansion.^44^ In contrast, the HS region displays an anomalous temperature dependence. As shown in Figure 5b, the PL intensity exhibits a non-monotonic trend: it initially increases upon cooling from room temperature, reaching a maximum at approximately 220 K, and subsequently decreases upon further cooling to 120 K.

**Figure 5:**
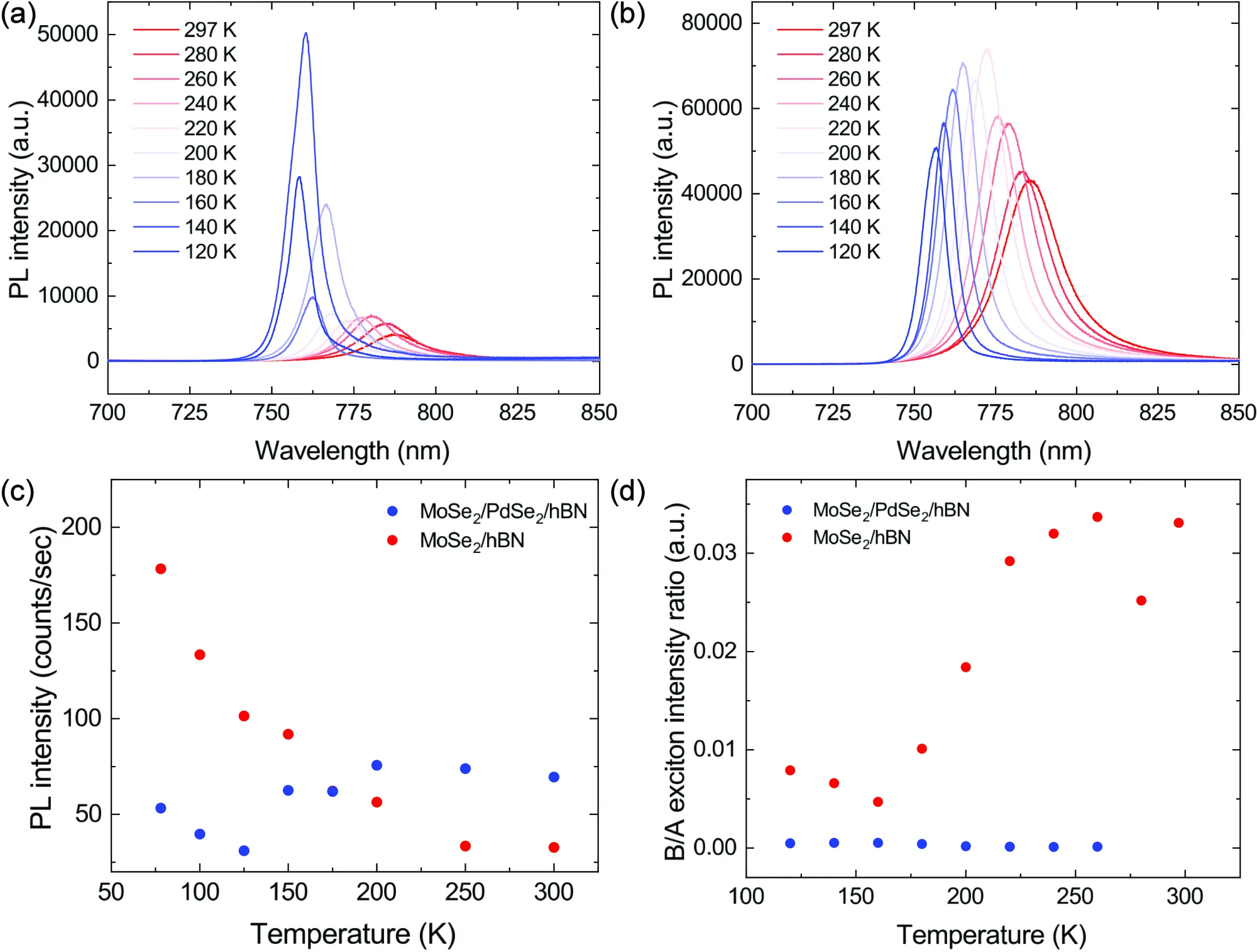
Temperature-dependent PL measurements of (a) the 1L region and (b) the HS region. The normalized PL spectra are shown in Figure S10. (c) Temperature-dependent peak PL measurements of the 1L region and the HS region. (d) Temperature dependence of the 1L region and the HS region B/A-exciton intensity ratio.

By comparison, the 1L A-exciton PL increases monotonically with a decreasing temperature (Figure 5c). This contrasting behavior demonstrates that PdSe_2_ modifies exciton relaxation dynamics in MoSe_2_. This is directly supported by time-resolved (TR) transient reflectivity measurements (Figure S11), which reveal a delayed, picosecond time scale rise of the A-exciton transient signal in the HS that is absent in isolated 1L MoSe_2_, providing direct kinetic evidence for interlayer carrier transfer from PdSe_2_ to MoSe_2_ that supplements the A-exciton population. Independent of this early time process, the reduced PL intensity in the HS region below ∼200 K reflects a temperature-dependent change in the balance of radiative and non-radiative recombination processes. This behavior is consistent with reduced phonon-assisted scattering,^47^ which can limit dark–bright exciton conversion and intervalley relaxation,^61,62^ thereby reducing the repopulation of the radiative A-exciton state. This behavior is hence not governed by EEA effects. To further probe the energetic origin of this behavior, we extracted the peak energies of the A and B excitons as a function of the temperature in both regions and calculated the effective spin–orbit splitting Δ*E*_B–A_ (Figure S12).

Figure 5d shows the temperature-dependent intensity ratio of the B exciton to the A exciton. In the 1L region, the ratio exhibits an overall increasing trend, reaching approximately 0.03 at 300 K, consistent with exciton thermalization governed by Boltzmann statistics.^47^ In contrast, the HS B exciton is only resolvable below ∼260 K, where the B/A ratio remains confined to a low range and shows only a weak temperature dependence compared to the clear Boltzmann-like increase in the 1L region. This behavior is consistent with a modification of the B-exciton thermalization dynamics in the presence of the PdSe_2_ interface.

To further probe how interlayer interactions affect exciton generation and recombination, we performed photoluminescence excitation (PLE) spectroscopy on the HS. Unlike conventional PL measurements, PLE examines how the excitation energy influences the emission intensity, providing insight into absorption pathways, exciton relaxation, and interlayer coupling.^63^ PLE spectroscopy reveals a consistent broadband PL enhancement of emission from the HS region across the entire excitation energy range compared to the 1L region (Figure 6a). Although excitation near 700 nm, corresponding to the MoSe_2_ B-exciton resonance, produces the highest absolute PL intensity due to stronger intrinsic absorption, the PL enhancement persists across the full spectral range (∼450–725 nm) rather than being confined to a specific transition. The wavelength-dependent PL enhancement factor extracted from the PLE spectra (Figure 6b) reveals the largest PL enhancement at shorter excitation wavelengths, exceeding ∼3.5 near 450 nm while decreasing to approximately ∼1.5 in the ∼550–650 nm range but remaining greater than unity. Unlike the PL quenching across the whole range observed in MoS_2_/PdSe_2_ (Figure S13), the MoSe_2_-based HS exhibits a divergent excitation response, demonstrating that interfacial band alignment dictates the transition between emission enhancement and suppression.

**Figure 6:**
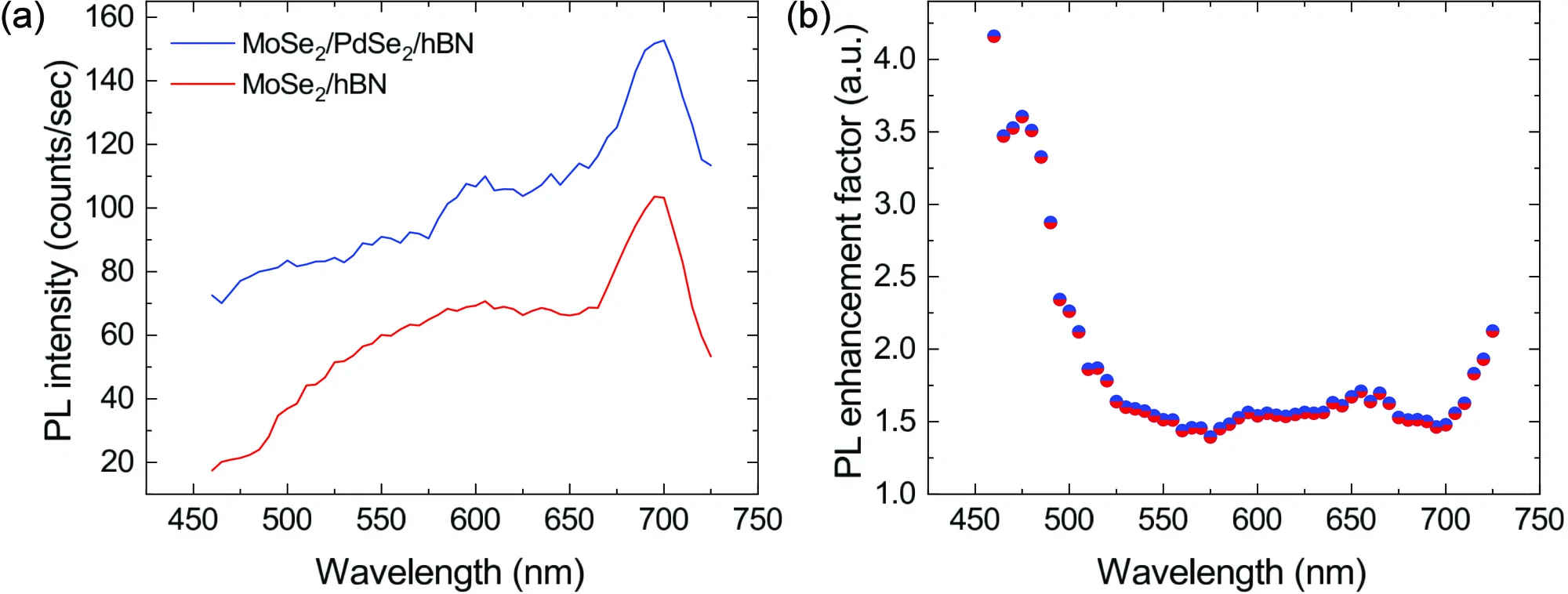
(a) PLE spectra of the 1L and the HS. (b) PLE intensity ratio between the HS and the 1L.

In this work, we have shown that interlayer coupling in MoSe_2_/PdSe_2_ HSs enables strong enhancement of A-exciton emission in 1L MoSe_2_, increasing the PLQY from ∼1% in the reference 1L to 6% at room temperature. Our method exploits interlayer coupling in a fully assembled heterostructure, enhancing emission while preserving the intrinsic properties of the 1L. The enhancement is accompanied by pronounced B-exciton quenching, reduced EEA, and a broadband increase in excitation efficiency. The observation that emission is enhanced in the wider bandgap layer, together with the broadband PLE response and temperature-dependent crossover to quenching, indicates that the effect does not arise from simple exciton funneling or a single-resonant process. Instead, the combined PL, broadband excitation PLE measurements, temperature-dependent PL, and DFT calculations indicate a mechanism in which interlayer electronic interactions redistribute exciton populations and modify relaxation pathways.

These findings establish interlayer coupling as a means to control exciton dynamics and enhance radiative recombination in two-dimensional semiconductors, offering a general strategy for improving light emission in vdW HSs. The enhanced A-exciton PL in MoSe_2_/PdSe_2_ arises from favorable interlayer coupling, which requires two criteria: (i) the 1L VBM lies near a high-density-of-states region of the narrow-gap substrate, enabling hybridization (the ∼100 meV offset in MoSe_2_/PdSe_2_ meets this, while MoS_2_/PdSe_2_ does not), and (ii) the substrate has a low density of states at the A-exciton energy, preventing excessive transfer and allowing radiative recombination to dominate. Because these criteria depend on band alignment and density of states, which can be tuned through material selection and stacking in a wide range of vdW HSs, they open opportunities for systematic design of materials with controlled exciton dynamics and improved light emission.

## Supplementary Material



## Notes

Experimental section, Raman spectroscopy, AFM topography, lattice parameter optimization, electronic structure calculations, quantifying interlayer hybridization, electronic structure and PL quenching in MoS_2_/PdSe_2_ heterostructures, PL spectra under 633 nm excitation, temperature-dependent evolution of excitonic emission, time-resolved measurement, temperature-dependent A- and B-exciton energies and effective spin–orbit splitting, and PLE spectra of MoS_2_-based samples (PDF)
