# Supplementary material for: Highly Efficient Exciton Modulation in MoSe2/PdSe2 Heterostructures
Source: Nano Lett. 2026 Sep 23;26(39):13292–300. doi: 10.1021/acs.nanolett.6c02581 (PMC13651624; doi:10.1021/acs.nanolett.6c02581)
Supplement: Supplementary file 1 [file nl6c02581_si_001.pdf]

Highly Efficient Exciton Modulation in $MoSe_2/PdSe_2$ Heterostructures

Petr Rozhin[1], Muhammad Sufyan Ramzan[2], Emma Contin[1], Danae Katrisioti[3,4], Till Weickhardt[5], Micol Bertolotti[6], Nouha Loudhaief[1], Bing Wu[7], Zdeněk Sofer[7], Takashi Taniguchi[8], Kenji Watanabe[9], Leonardo Puppulin[1], Caterina Cocchi[2], Ioannis Paradisanos[3,4], Giancarlo Soavi[5], Stefano Dal Conte[6], Giovanni Antonio Salvatore[1], and Domenico De Fazio*[1]

[1]Department of Molecular Sciences and Nanosystems, Ca' Foscari University of Venice, 30172 Venice, Italy
[2]Institute of Condensed Matter Theory and Optics, Friedrich Schiller University Jena, Jena 07743, Germany
[3]Department of Materials Science and Engineering, University of Crete, Heraklion, 70013 Crete, Greece
[4]Institute of Electronic Structure and Laser, Foundation for Research and Technology-Hellas, Heraklion, 70013 Crete, Greece
[5]Institute of Solid State Physics, Friedrich Schiller University Jena, Jena 07743, Germany
[6]Department of Physics, Politecnico di Milano, I-20133 Milano, Italy
[7]Department of Inorganic Chemistry, University of Chemistry and Technology Prague, 166 28 Prague 6, Czech Republic
[8]Research Center for Materials Nanoarchitectonics, National Institute for Materials Science, 1-1 Namiki, Tsukuba 305-0044, Japan
[9]Research Center for Electronic and Optical Materials, National Institute for Materials Science, 1-1 Namiki, Tsukuba 305-0044, Japan

*Email: domenico.defazio@unive.it

Supporting Information

Experimental section

Crystal growth: Orthorhombic $PdSe_2$ was made by flux growth in a quartz ampoule using Pd and Se. The stoichiometric amount of Pd (powder sponge, 99.95%, Safina, Czech Republic) and selenium (2-4 mm granules, 99.9999%, Wuhan Xinrong New Materials Co., China) were placed in stoichiometric amount corresponding to 3 g in quartz ampoule (25 x 100 mm^2) and melt sealed by oxygen-hydrogen welding torch under high vacuum ($<1{\cdot}10^{-3}$ Pa, oil diffusion pump with LN_2 trap). The ampoule was placed in a muffle furnace and heated at 850 °C (heating rate 1 °C/min), and after 5 hours, cooled to 500 °C using a cooling rate of 6 °C/hour and finally freely cooled to room temperature. Ampoules were opened in an argon-filled glovebox, and crystals were collected. 2H-$MoSe_2$ was made by the chemical vapor transport (CVT) method using bromine as a transport medium. Selenium (2-4 mm granules, 99.9999%, Wuhan Xinrong New Materials Co., China) and molybdenum (-100 mesh, 99.95%, Molymet, Germany) were placed in ampoule (40 x 250 mm^2) with stochiometric ratio corresponding to 30 g of $MoSe_2$ together with 1 g of $SeBr_4$ (99%, Strem, USA) and melt sealed by oxygen-hydrogen welding torch under high vacuum ($<1{\cdot}10^{-3}$ Pa, oil diffusion pump with LN_2 trap). The ampoule was placed in a muffle furnace and heated at 500 °C for 25 hours, at 600 °C for 50 hours,

and finally at 800 °C for 50 hours. The heating and cooling rates were 1 °C/min. The ampoule was placed in a two-zone furnace. First, the growth zone was heated to 1000 °C and the source zone to 800 °C, and after 2 days, the thermal gradient was reversed, and the growth zone was heated to 900 °C and the source zone to 1000 °C for 14 days. Finally, the ampoule was cooled to room temperature and opened in an argon-filled glovebox.

HS preparation: 1L $MoSe_2$ (and MoS_2), multilayer $PdSe_2$, and hBN flakes were mechanically exfoliated from their respective bulk crystals. The HSs $MoSe_2$/$PdSe_2$/hBN (and MoS_2/$PdSe_2$/hBN) were fabricated on SiO_2/Si substrates using a standard dry transfer method with a polydimethylsiloxane (PDMS) stamp.[S1] Using an optical microscope and an aligned manual transfer system (HQ Graphene), the layers were successively transferred onto the substrate in the following sequence: bottom hBN, $PdSe_2$ multilayer, and finally the $MoSe_2$ (or MoS_2) 1L.

AFM characterization: To probe the surface topography and determine the layer thickness, atomic force microscopy (AFM) measurements were performed at room temperature using a Bruker Dimension Icon instrument (Bruker Corporation, Billerica, MA, USA) and the ScanAsyst-Air cantilever (Bruker Corporation, Billerica, MA, USA) with a resonance frequency of 70 kHz, a spring constant of 0.4 N/m, and a nominal tip radius of 2 nm. Height images of the HS region, the 1L region, and $PdSe_2$ flakes were acquired using the PeakForce Tapping mode, operating at the off-resonance frequency of 2 kHz. Force set-point and scan rate were set to 0.5 nN and 0.6 Hz, respectively.

Computational methods: All calculations were performed within the framework of DFT[S2] using the Vienna ab-initio simulation Package (VASP).[S3] The electron-ion interactions in the Kohn-Sham equations[S4] were treated with the projector augmented-wave method,[S5] and the exchange-correlation effects were described with the Perdew, Burke, and Ernzerhof functional[S6] within the generalized gradient approximation. vdW interactions were included through the optPBE-vdW density-functional scheme,[S7] which provides the most accurate description of the lattice parameters of multilayer $PdSe_2$.[S8] A plane wave energy cutoff of 500 eV was employed, and the Brillouin zone was sampled using a $12 \times 12 \times 1$ k-point mesh. Spin-orbit coupling was included in all electronic structure calculations; in these cases, the increased computational cost was mitigated by reducing the energy cutoff to the `ENMAX` value of the employed pseudopotentials. The self-consistent field and structural relaxation were converged to 10^{-6} eV for the total energy and 15 meV Å^{-1} for the residual forces. To eliminate artificial interactions between periodic images, a 16 Å vacuum region was introduced perpendicular to the 1L plane. The crystal structures were visualized using `ASE`,[S9] and the post-processing code `SUMO`[S10] was used to plot the band structures and the PDOS.

Photoluminescence and Raman measurements: PL and Raman measurements were performed using a Renishaw inVia confocal Raman microscope (Renishaw, UK). High-frequency Raman spectra were acquired with a 514 nm excitation laser at 0.05 mW through a 100× objective, dispersed by a 1800 lines/mm holographic grating. Low-frequency Raman measurements used the same laser at 0.05 mW, with an ECLIPSE filter and a 2400 lines/mm grating, 300 s exposure, and a 100× objective. PL spectra were recorded using 514 nm or 633 nm excitation at 0.05 mW and 0.075 mW, dispersed by a 1200 lines/mm grating with 10 s exposure through a 100× objective. PL mapping employed a 514 nm laser at 0.5 mW with a 1200 lines/mm grating, 10 s exposure per pixel, and a 100× objective, with spatial maps constructed from the A-exciton peak intensity at 786 nm and the B/A exciton intensity ratio (702/786 nm). Temperature-dependent PL was performed using a Linkam HFS600E-PB4 heating/cooling stage, exciting the sample with a 514 nm laser (0.05 mW) through a long-working-distance 50× objective, dispersed by a 1200 lines/mm grating with 10 s exposure. Power-dependent PL measurements at room temperature were performed using the same Renishaw inVia confocal Raman microscope by varying the excitation power of the 514 nm laser. Power-dependent PL measurements at cryogenic temperatures, along with temperature-dependent PL studies, were performed using a custom-built micro-spectroscopy setup that includes a continuous-flow cryostat (Janis ST-500). Mechanical drifts associated with the cryogenic system are likely responsible for the slight PL intensity fluctuations as a function of the excitation power; however, the impact on the extracted power-law exponents is minimal. The emitted signal was dispersed by a Horiba iHR-320 monochromator and detected using a thermoelectrically cooled CCD camera (Syncerity).

PLE: Room-temperature (295 K) photoluminescence excitation (PLE) measurements of the $MoSe_2$/hBN and $MoSe_2$/$PdSe_2$/hBN HSs were performed using a custom-built micro-spectroscopy setup. The samples were mounted on a Thorlabs PT3 XYZ mechanical stage using silver paste to ensure position control and mechanical stability. Excitation was provided by a tunable supercontinuum white-light laser (NKT Pho-

tonics) and focused onto the sample through a Mitutoyo 50×/0.42 NA objective, resulting in a spot size of approximately 1 μm. The excitation wavelength was tuned from 450 nm to 725 nm in steps of 5 nm, while the incident laser power was maintained constant at 10 μW across the entire spectral range. To suppress laser scattering, a 775 nm short-pass filter was used in the excitation path, while a corresponding 775 nm long-pass filter was placed in the collection path (both from Edmund Optics). All spectra were acquired with an exposure time of 5 s and a single accumulation for each excitation wavelength.

Raman spectroscopy

Figure S1 presents the Raman spectra acquired from three regions: $PdSe_2$ on hBN, the 1L region, and the HS region. Low-frequency Raman spectroscopy (0–100 cm^{-1}) is a widely used, non-destructive probe of interlayer vibrational modes in two-dimensional materials. For $PdSe_2$, the corrected linear chain model[S11] can be used to estimate the number of layers from the peak positions of shear (SM) and layer-breathing (BM) modes via

$$\omega(B_j) \approx \sqrt{2}\,\omega(L, B_1)(1-\beta)\left[\sin^2\left(\frac{j\pi}{2N}\right) + \beta\sin^2\left(\frac{j\pi}{N}\right)\right]^{1/2} \tag{S1}$$

where $\omega(B_j)$ is the Raman shift (in cm^{-1}) of the j-th BM branch, with $\omega(L, B_1)$=43.2 cm^{-1}, β=0.13, and N is the number of layers. From this analysis, the extracted layer number of $PdSe_2$ is approximately six layers. The identification of the 1L region is supported by the absence of low-frequency Raman peaks, as these modes originate from interlayer vibrations. In the high-frequency range (100-350 cm^{-1}), the 1L region exhibits two characteristic Raman peaks corresponding to the $A_1^{'}$ (242 cm^{-1}) and $E^{'}$ (287 cm^{-1}) modes. In contrast, $PdSe_2$ shows four distinct peaks associated with the A_g^1 (146 cm^{-1}), A_g^2 (209 cm^{-1}), B_{1g}^2 (225 cm^{-1}), and A_g^3 (261 cm^{-1}). In the HS region, all $PdSe_2$ peaks remain clearly visible, whereas the Raman intensities of the $MoSe_2$ modes are suppressed, indicating an interaction between the two layers.

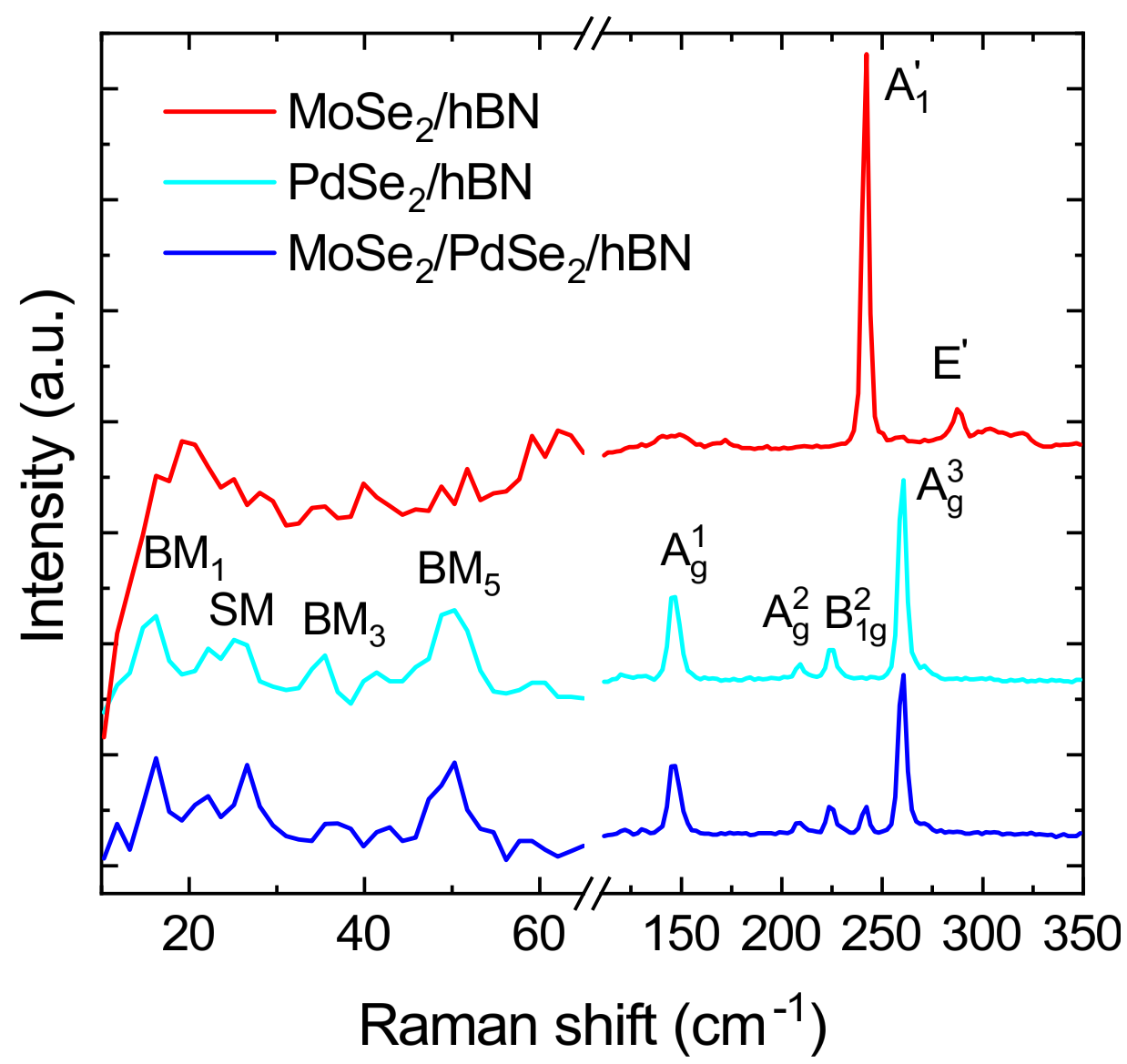

Figure S1: Raman spectra acquired from the 1L region, $PdSe_2$ on hBN, and the HS region.

AFM topography

AFM was used to verify the thickness and vertical stacking of the $MoSe_2/PdSe_2$ HS on hBN. The topography map of the measured region is shown in Figure S2(a), where terraces corresponding to the different constituent layers are clearly visible. The height profile extracted along the green/magenta line in Figure S2(a) (profile in Figure S2(b)) traverses a region without overlap between $PdSe_2$ and $MoSe_2$, providing an accurate measurement of the 1L $MoSe_2$ thickness. Along the scan, the first terrace corresponds to 1L $MoSe_2$ on hBN, with a step height of ∼0.8 nm above the hBN surface. This is followed by the bare hBN region (∼22.4 nm), forming the lowest terrace, and then a multilayer $PdSe_2$ region (∼7.7 nm above hBN). The profile then returns to the hBN-only baseline. This scan is therefore useful for precisely quantifying the 1L $MoSe_2$ thickness and verifying the $PdSe_2$ step height, confirming the vertical dimensions of the individual constituents.

A separate line profile along the path shown in Figure S2c traverses the $PdSe_2$ region and captures the HS. The lowest terrace corresponds to bare hBN (∼26.3 nm), followed by a multilayer $MoSe_2$ region on hBN (∼27.7 nm) and a 1L $MoSe_2$ on hBN (∼26.4 nm). The overlapping HS region, where $MoSe_2$ sits on top of $PdSe_2$, exhibits a height of ∼37.9 nm, consistent with the sum of the individual layers, and displays a relatively smooth surface with an RMS roughness below 1.4 nm. The next terrace corresponds to the $PdSe_2$ multilayer on hBN (∼34.1 nm). Notably, low-frequency Raman measurements indicate that this $PdSe_2$ flake consists of six layers, whereas the AFM-measured step height of ∼7.7–8.5 nm is slightly larger than the expected crystallographic thickness for six layers, a discrepancy commonly observed in AFM measurements of 2D materials due to tip-sample interactions, adsorbates, or surface roughness.[S12,S13] The scan then returns to the bare hBN baseline (∼25.6 nm). Together, these profiles confirm the vertical stacking hierarchy of the HS and provide direct thickness measurements consistent with Raman-based layer assignment.

To assess whether the transfer process introduced appreciable strain, we examined the HS region by AFM and high-frequency Raman spectroscopy. The in-plane $E^{'}$ mode of $MoSe_2$, most sensitive to mechanical strain, is significantly suppressed in intensity within the HS region (Figure S1), precluding a reliable comparison of its peak position relative to the 1L region. We therefore assess residual strain through AFM topography and structural considerations. The smooth surface morphology and the absence of visible wrinkle-like features (Figure S2) suggest that transfer-induced strain is minimal. In addition, the distinct crystal symmetries and lattice periodicities of $MoSe_2$ (hexagonal) and $PdSe_2$ (orthorhombic) preclude commensurate registry between the two lattices, further limiting the likelihood of coherent interfacial strain accumulation or reconstruction. Although transfer-induced strain has been reported in mechanically assembled van der Waals HSs,[S14,S15] our AFM measurements, together with this structural incommensurability, indicate that strain effects in the present HSs are minor and unlikely to play a significant role in the observed optical response.

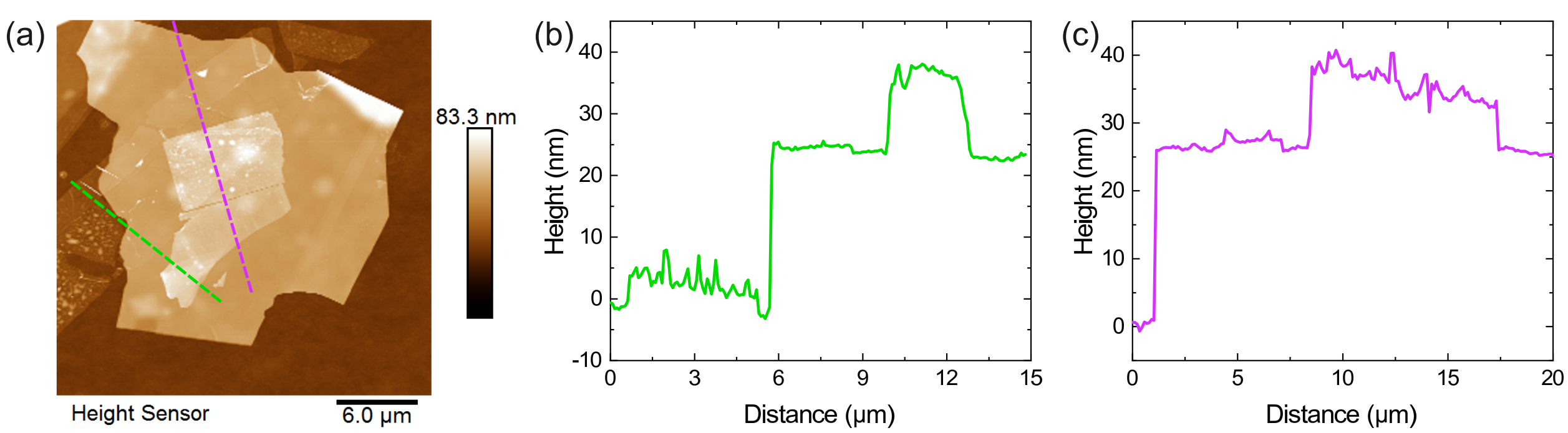

Figure S2: (a) AFM topography of the measured area of the $MoSe_2/PdSe_2$ HS on hBN showing regions with different thicknesses; (b, c) corresponding height profiles taken along the green and magenta dashed lines in (a).

Lattice parameters optimization

In our simulations, the 4L $PdSe_2$ and the 1L MoS_2 and 1L $MoSe_2$ were taken from their orthorhombic and 2H bulk phases, respectively. The relaxed in-plane lattice parameters of the pure layers are: a = 5.820 Å, b = 5.974 Å for 4L $PdSe_2$, a = b = 3.166 Å for 1L MoS_2, and a = b = 3.298 Å for 1L $MoSe_2$, in good agreement with the previous reports,[S8,S16,S17] confirming the accuracy of our adopted simulation parameters. The HSs were built by stacking a 2×2 rectangular supercell of MoS_2 (or $MoSe_2$) on a 1×1 cell of 4L $PdSe_2$ unit cell, resulting in 36 atoms per simulation cell, as shown in Figure S3. For the $MoSe_2$/$PdSe_2$ HS, the energetically most favorable AB stacking configuration was adopted.[S18] The rectangular supercell of the TMD layer was chosen to match the orthorhombic $PdSe_2$ unit cell while minimizing the in-plane strain arising from the lattice mismatch. The resulting simulated HSs therefore correspond to untwisted interfaces. The in-plane lattice vectors and atomic positions were fully relaxed using vdW-corrected DFT (see Computational methods) to diffuse the lattice mismatch between layers. The final relaxed lattice parameters are a = 5.842 Å and b = 6.250 Å for 1L $MoSe_2$/4L $PdSe_2$, and a = 5.721 Å and b = 6.132 Å for 1L MoS_2/4L $PdSe_2$. The resulting interlayer distance between the topmost Se atom of 4L $PdSe_2$ and the nearest Se and S atoms of the 1L $MoSe_2$ and 1L MoS_2 is 3.11 Å and 3.04 Å, respectively. These vdW gaps obtained after full relaxation are within the typical range reported for TMD-based HSs.[S16]

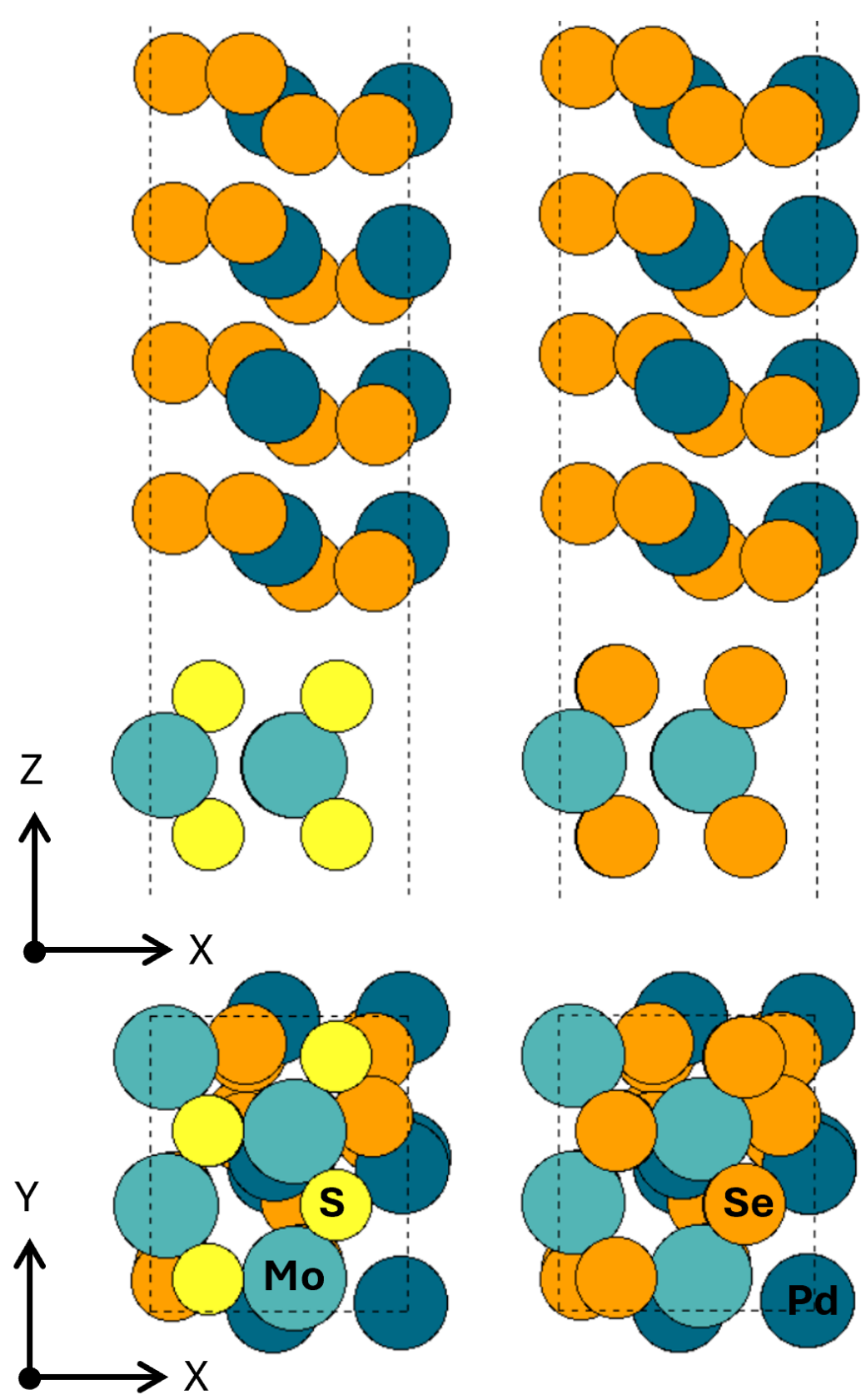

Figure S3: Top and side view of the crystal structure of 1L MoS_2 and 1L $MoSe_2$ stacked on 4L $PdSe_2$.

Electronic structure calculations

To investigate the influence of the vdW interface on the electronic properties, *ab initio* simulations were performed. As shown in Figures S4(a,b), the calculated electronic band structures of 4L and 6L $PdSe_2$ exhibit qualitatively analogous electronic features. Consequently, 4L $PdSe_2$ was utilized as a computationally efficient proxy for the experimental 6L $PdSe_2$ in the HS simulations.

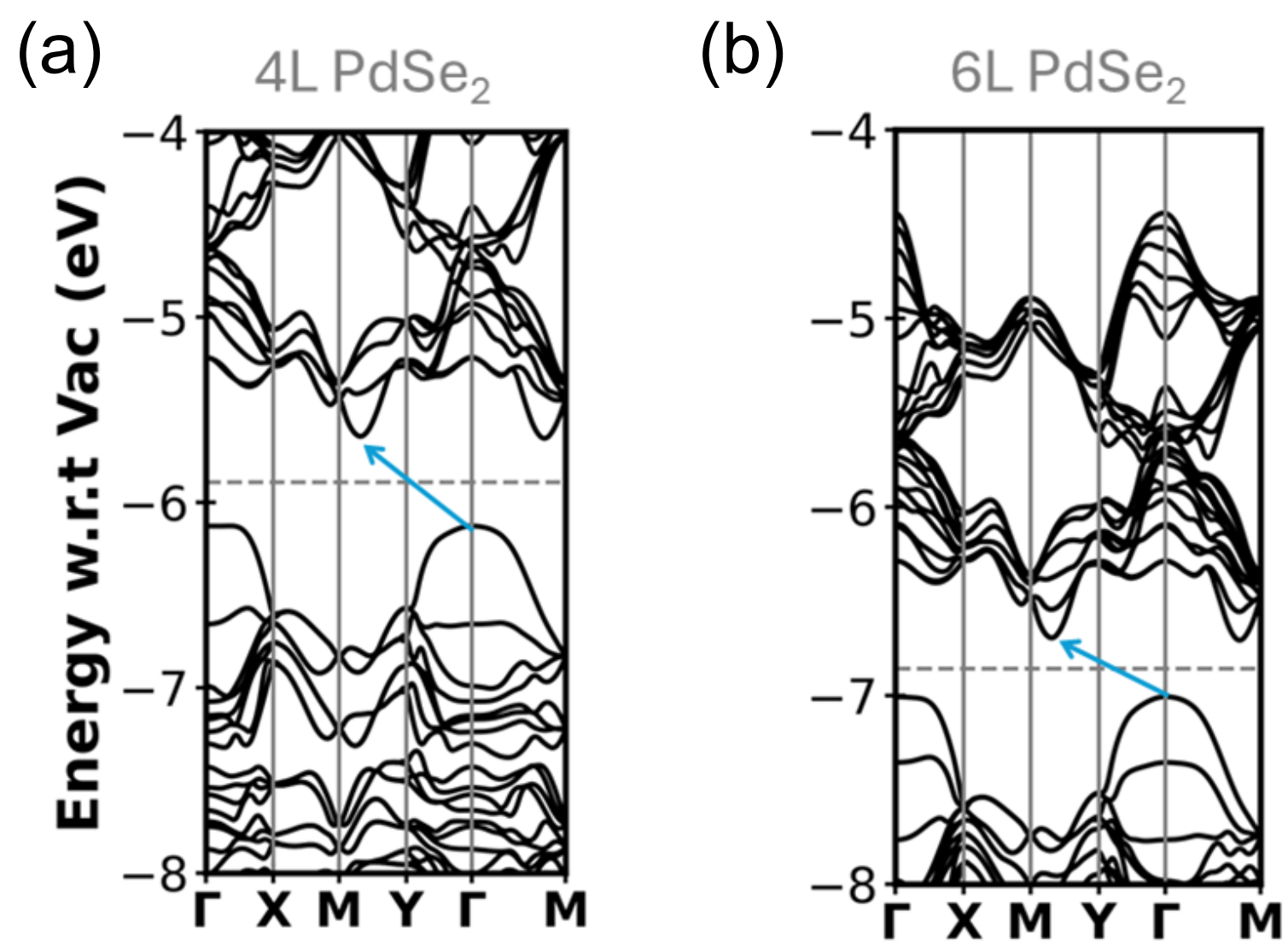

Figure S4: Electronic band structure of (a) 4L- and (b) 6L-$PdSe_2$ calculated with opt-PBE vdW functionals. The vacuum level is set to zero, and the blue arrows mark fundamental gaps. The Fermi-level marked with a gray-dashed line is set to the mid band gap.

The minor differences in the electronic band structures between the 4L and 6L systems were calculated and shown in Table S1.

System	Vacuum level (eV)	Work function (eV)	Electron affinity (eV)	Ionization energy (eV)
4L-$PdSe_2$	5.891	5.129	4.708	5.131
6L-$PdSe_2$	6.587	5.015	4.766	5.018

Table S1: Calculated vacuum level, work function, electron affinity, and ionization energy for the 4L and 6L $PdSe_2$.

Despite the presence of layer-dependent quantum confinement in multilayer $PdSe_2$, we found that the 4L $PdSe_2$ system closely reproduces the electronic environment of the experimentally adopted 6L $PdSe_2$ (Figure S4, Table S1). In particular, the band-edge positions and work functions differ by less than ∼0.1 eV (Table S1), indicating that the interlayer hybridization mechanism is not expected to be significantly affected by the reduced $PdSe_2$ thickness.

Quantifying interlayer hybridization

To quantify the strength of interlayer electronic hybridization by the orbital-projected band structures (Figure 1b), we analyzed the real-space probability densities of the valence-band states at the K-valley that give rise to the A- and B-excitons (Figure S5). The calculations reveal that while the wavefunction associated with the A-valley remains strictly localized within the monolayer (1L) $MoSe_2$, the B-state wavefunction significantly leaks into the adjacent $PdSe_2$ layers, showing a pronounced contribution from the interfacial Se atoms. This spatial delocalization provides direct evidence of enhanced interlayer coupling for the B-valley states, which facilitates new relaxation pathways and drives the selective quenching of the B-excitonic resonance in the HS. In contrast, in the $MoS_2/PdSe_2$ control HS, the wavefunctions of both the A- and B-valleys remain almost completely confined to the MoS_2 layer, showing negligible spilling onto the $PdSe_2$ layers, a signature of much weaker interlayer hybridization.

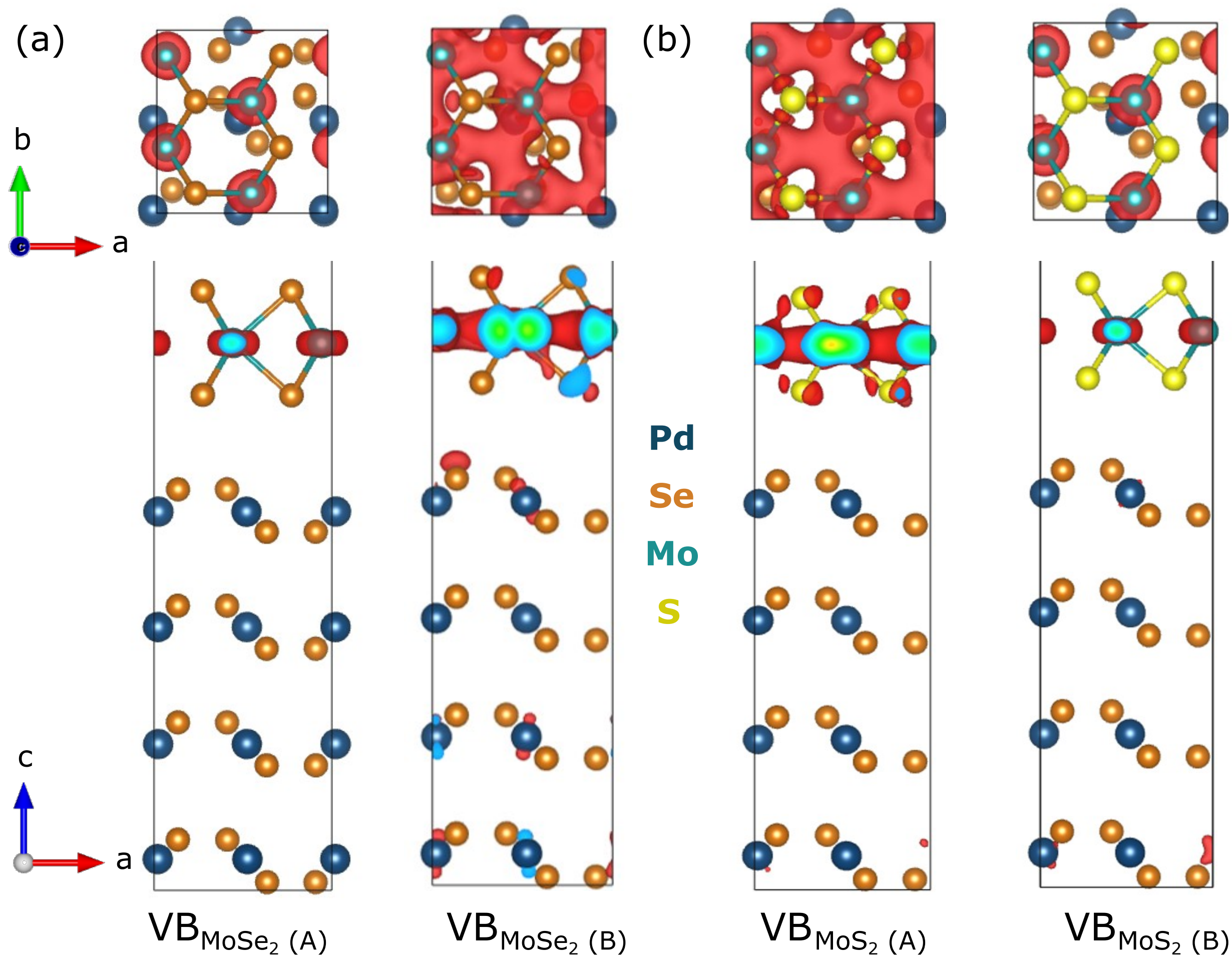

Figure S5: Real-space probability densities of the folded valence-band states at the high-symmetry point K, giving rise to the A and B excitons for the (a) $MoSe_2/PdSe_2$ and (b) $MoS_2/PdSe_2$ HSs.

To validate this interpretation, we examined the layer-resolved PDOS of the HS near the band edges (Figure S6a). The valence states of the 1L $MoSe_2$ shift toward lower energies, reflecting interface hybridization. Moreover, they align with the first two prominent PDOS peaks of the multilayer $PdSe_2$, promoting interlayer excitation pathways. In contrast, control calculations on an analogous $MoS_2/PdSe_2$ HS reveal that the MoS_2 valence states remain energetically isolated, ∼1.0 eV below the dominant PDOS maxima of $PdSe_2$ (Figure S6b), preventing similar hybridization.

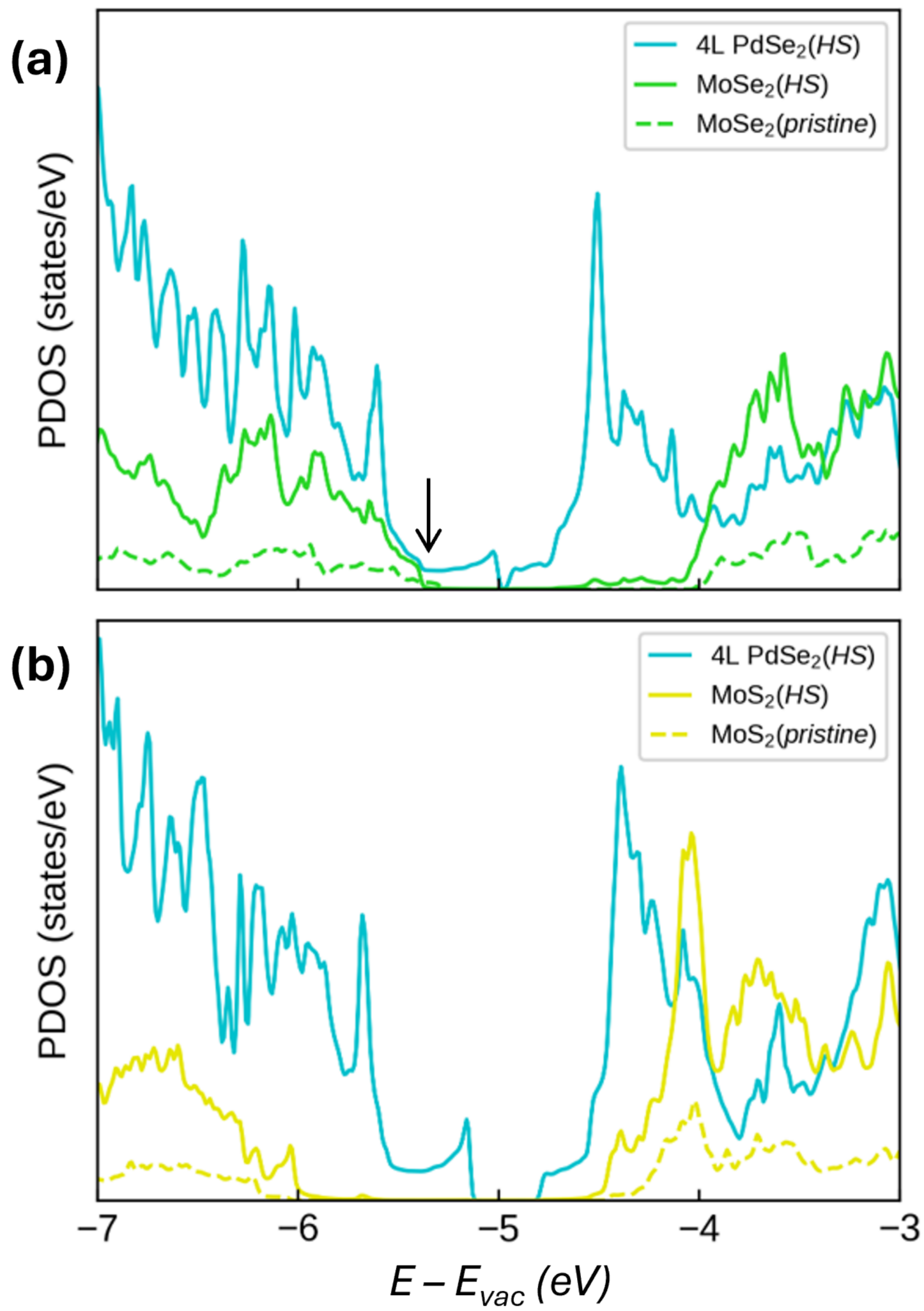

Figure S6: Layer-resolved projected density of states (PDOS) for the (a) $MoSe_2/PdSe_2$ and (b) $MoS_2/PdSe_2$ HSs, referenced to the vacuum level. The solid blue line represents the PDOS projected on the $PdSe_2$ layers, while the solid and dashed green ($MoSe_2$) and yellow (MoS_2) lines represent the PDOS of the 1L in the HSs and in their isolated forms, respectively.

To explicitly distinguish between electron and hole dynamics, the proposed microscopic relaxation pathways under resonant and non-resonant conditions are schematically illustrated in Figure S7. Under resonant excitation, photoexcitation is localized primarily within the 1L $MoSe_2$. While the A-exciton hole states remain localized within 1L $MoSe_2$, hybridization of the deeper B-valley valence states opens a non-radiative interlayer hole-relaxation channel into the $PdSe_2$ valence band, consistent with the selective suppression of B-exciton emission. Conversely, under non-resonant (high-energy) excitation, broadband absorption generates energetic ("hot") carriers in both layers. Because these high-energy photoexcited electrons and holes in $PdSe_2$ temporarily occupy states higher in energy than the band edges of 1L $MoSe_2$, they naturally transfer across the hybridized interface into the lower-energy band edges of $MoSe_2$. This two-carrier influx repopulates the localized A-exciton in $MoSe_2$, providing a physically sound rationale for the pronounced off-resonant PL enhancement. In contrast, in the MoS_2/$PdSe_2$ HS, the absence of valence-state hybridization, together with the significant energetic separation between the MoS_2 and $PdSe_2$ band edges (Figure S8a), is consistent with an exciton-funneling-like process in which carriers transfer from MoS_2 to $PdSe_2$, depleting the MoS_2 exciton population and producing the PL quenching (Figures S8b and S8c).

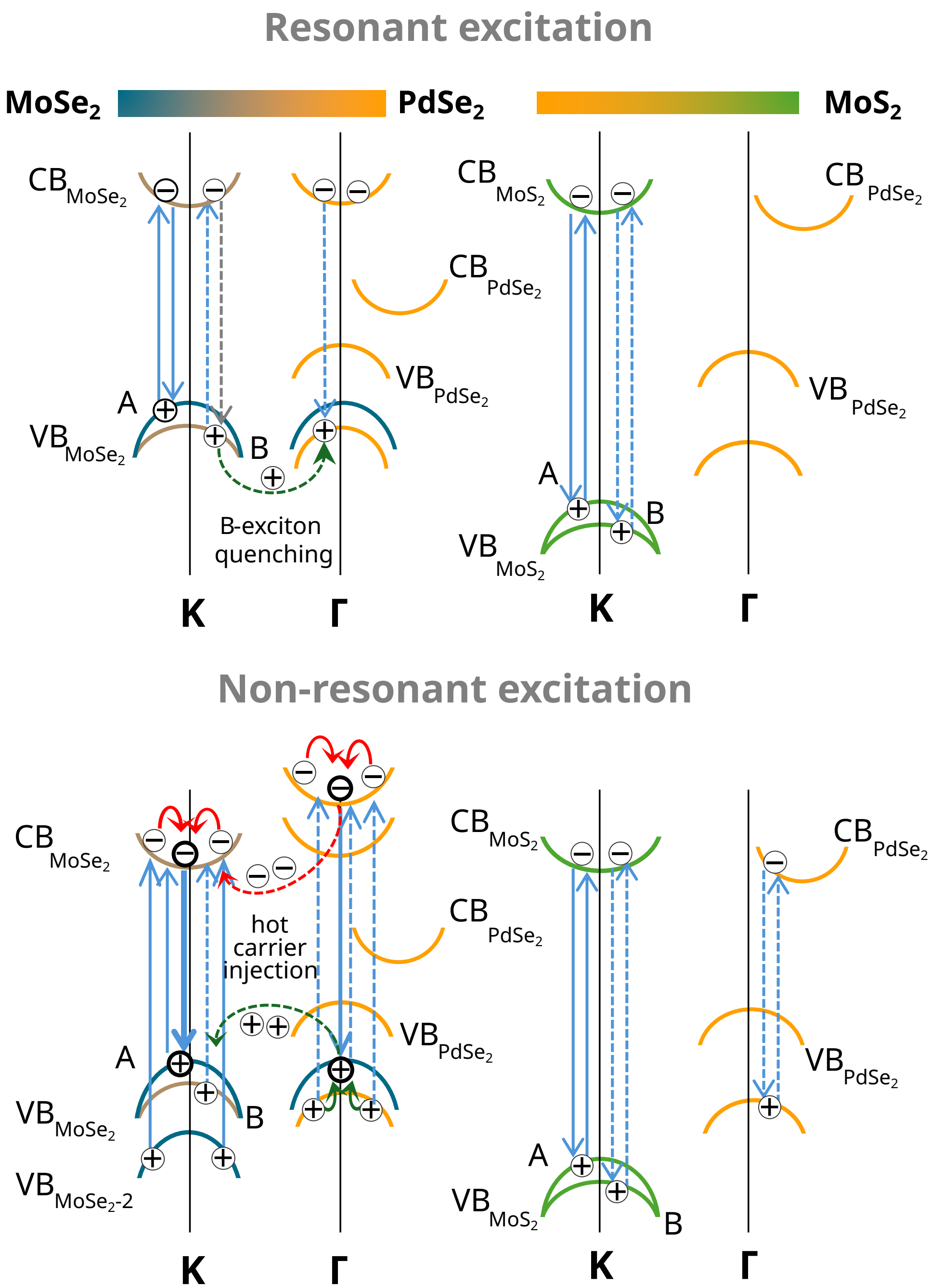

Figure S7: Schematic energy-level diagram illustrating the proposed carrier relaxation pathways in $MoSe_2/PdSe_2$ (left) and $MoS_2/PdSe_2$ (right) HSs under (top) resonant and (bottom) non-resonant excitation. Blue solid and dashed vertical arrows indicate optical absorption and radiative pathways associated with the A- and B- excitons, respectively. Encircled minus and plus signs denote electrons and holes, respectively. A grey dashed arrow denotes non-radiative recombination. Red arrows denote electron cooling/transfer pathways, while dashed green arrows denote hole relaxation/transfer pathways.

Electronic Structure and PL Quenching in $MoS_2/PdSe_2$ Heterostructures

Figure S8(a) illustrates the electronic band structure and PDOS for the 1L MoS_2/4L $PdSe_2$ HS. In contrast to the $MoSe_2$ case, the valence states of 1L MoS_2 (indicated by the red arrow) lie approximately 1 eV below the frontier states of the multilayer $PdSe_2$. This large energy offset facilitates efficient hole transfer from the MoS_2 to the $PdSe_2$ layer, leading to the strong PL quenching observed experimentally in the $MoS_2/PdSe_2$ control samples (Figure S8(b,c)).

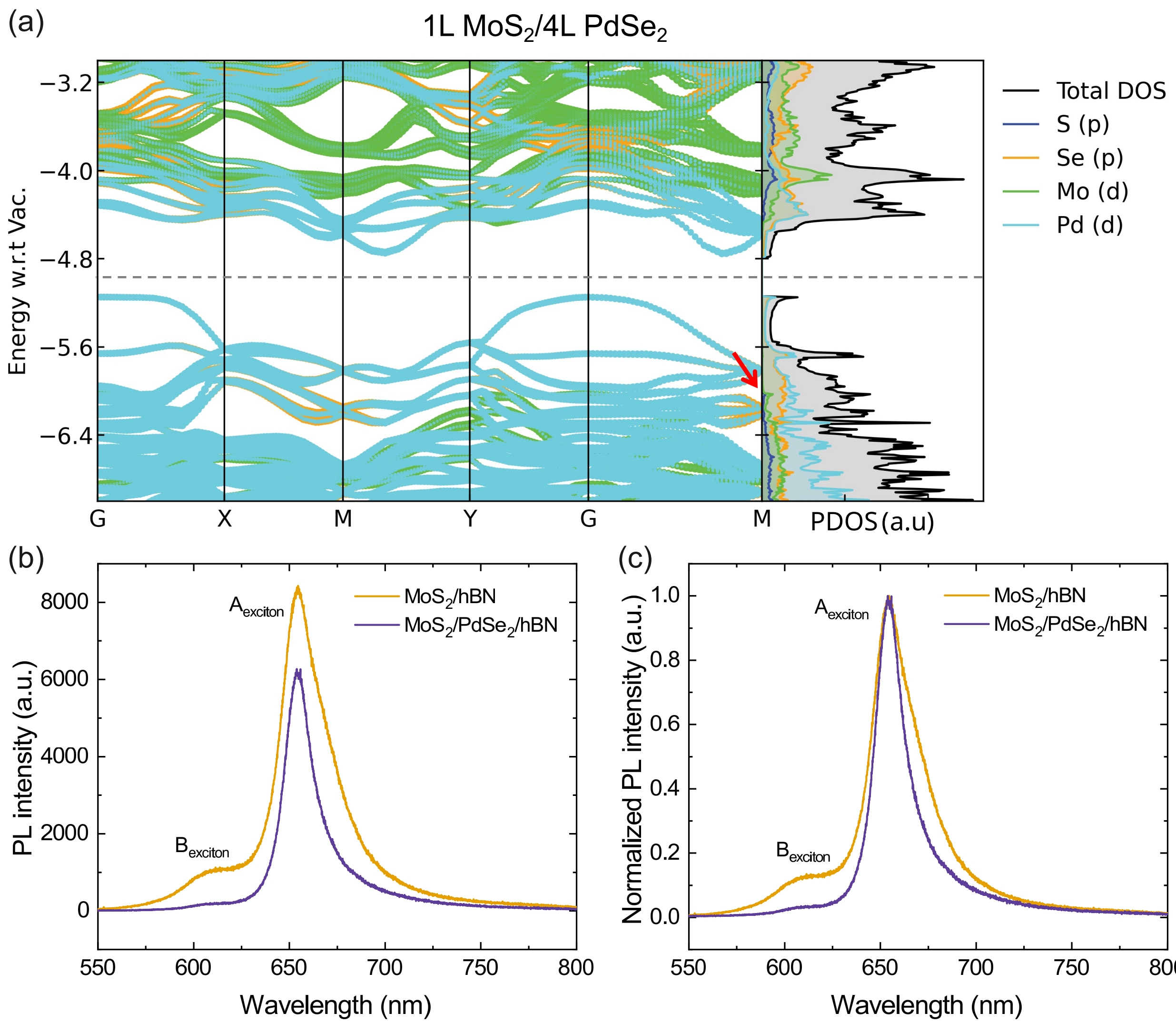

Figure S8: (a) Electronic band structures alongside atom PDOS of 1L MoS_2/4L $PdSe_2$. The vacuum level is set to zero, and the Fermi level marked with a horizontal dashed line is set to the midgap. The vertical grey line between high symmetry points Y and Γ corresponds to the folded high symmetry point K of the primitive hexagonal lattice. PL spectra of the 1L region and the HS region of MoS_2-based samples at 514 nm excitation (b) A-exciton quenching in the HS region (c) B-exciton quenching in the HS region.

PL spectra under 633 nm excitation

Figure S9 presents PL spectra of the 1L region and the HS region measured under 633 nm excitation. This measurement was performed to verify that the PL enhancement observed under 514 nm excitation is not specific to a single excitation wavelength. A clear enhancement of the A-exciton emission is again observed in the HS region, with the peak intensity increasing by approximately a factor of four relative to bare $MoSe_2$. The persistence of the enhancement under longer-wavelength excitation confirms that the effect is intrinsic to the HS region rather than arising from a particular excitation condition.

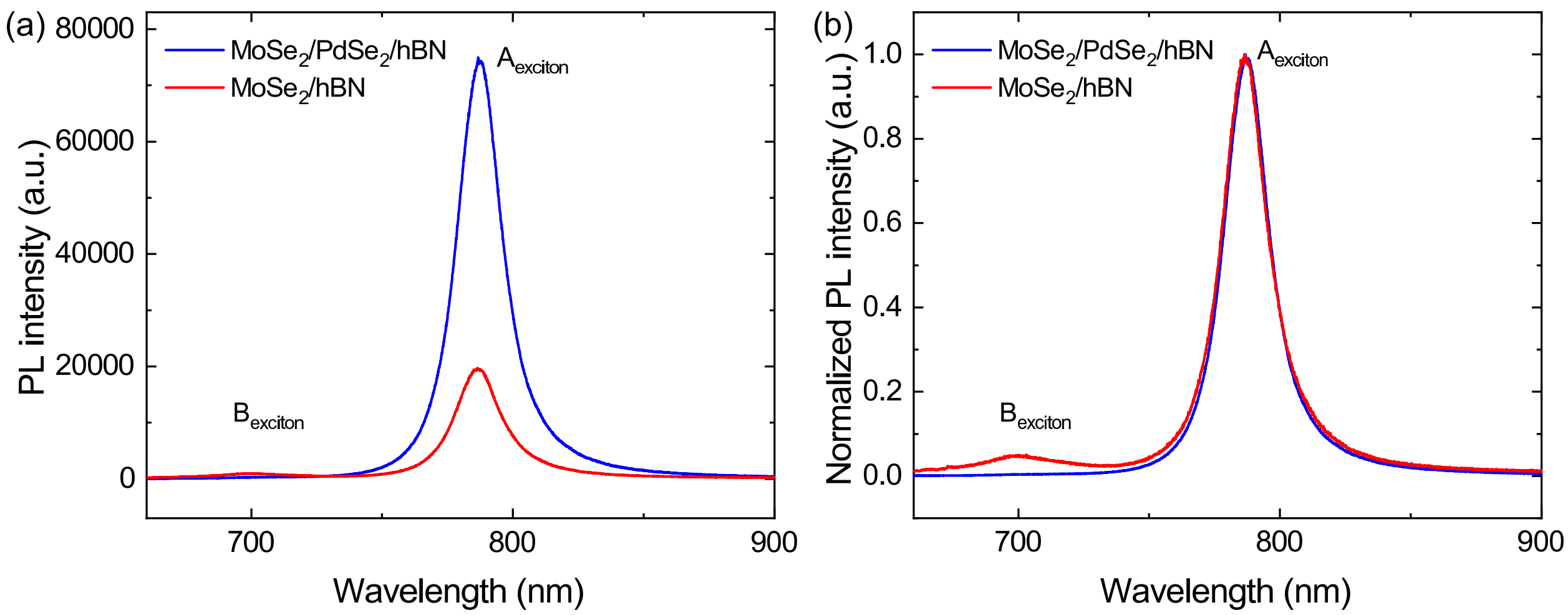

Figure S9: PL spectra of the 1L region and the HS region at 633 nm excitation (a) A-exciton PL enhancement in the HS region (b) B-exciton quenching in the HS region.

Temperature-dependent evolution of excitonic emission

Normalized PL spectra of the 1L region (Figure S10(a)) were measured from 297 K down to 120 K. With decreasing temperature, the A-exciton peak shows a progressive blueshift of approximately $\Delta E \approx 61$ meV and a reduction in full-width at half-maximum (FWHM) from ∼41 meV at 297 K to ∼16 meV at 120 K, consistent with the expected temperature dependence of the bandgap and reduced phonon-assisted broadening.[S19] The relative intensity of the B-exciton, quantified as the ratio I_B/I_A, decreases from ∼0.035 at 297 K to ∼0.008 at 120 K, likely due to enhanced B → A exciton intralayer relaxation and the stronger temperature dependence of the A-exciton radiative efficiency compared to the B-exciton.[S20]

For the HS region (Figure S10(b)), the A-exciton exhibits a blueshift and FWHM narrowing ($\Delta E \approx 60$ meV, from ∼42 meV at 297 K to ∼19 meV at 120 K), similar to the 1L region. In contrast, the B-exciton, which is absent at room temperature, gradually emerges at low temperatures (shown in the inset), reaching I_B/I_A ∼0.0005 at 120 K. This behavior reflects the interplay of competing channels: at room temperature, strong interlayer hybridization effectively quenches the B-exciton and enhances A-exciton emission, while partially blocking intralayer B → A relaxation. At low temperature, the hybridization weakens due to reduced phonon-mediated coupling, allowing a small fraction of B-excitons to survive and radiatively recombine. Even though B → A relaxation is intrinsically faster at low T, the residual hybridization and suppression of EEA at low powers enable the appearance of a weak B-exciton emission. Notably, the B-exciton in the HS region remains much weaker than in the 1L region, highlighting the persistent influence of interlayer hybridization on exciton dynamics.

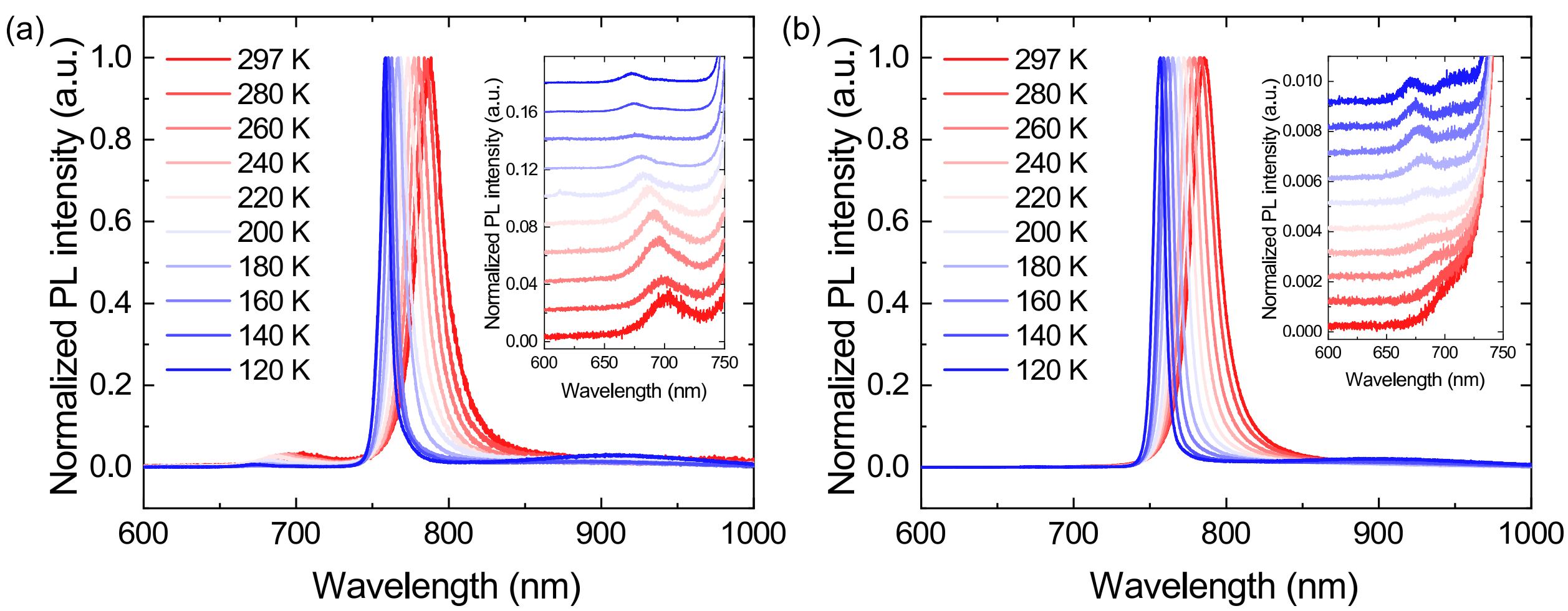

Figure S10: Temperature-dependent normalized PL spectra of (a) the 1L region and (b) the HS region. The insets highlight the B-exciton spectral region.

Time-resolved measurement

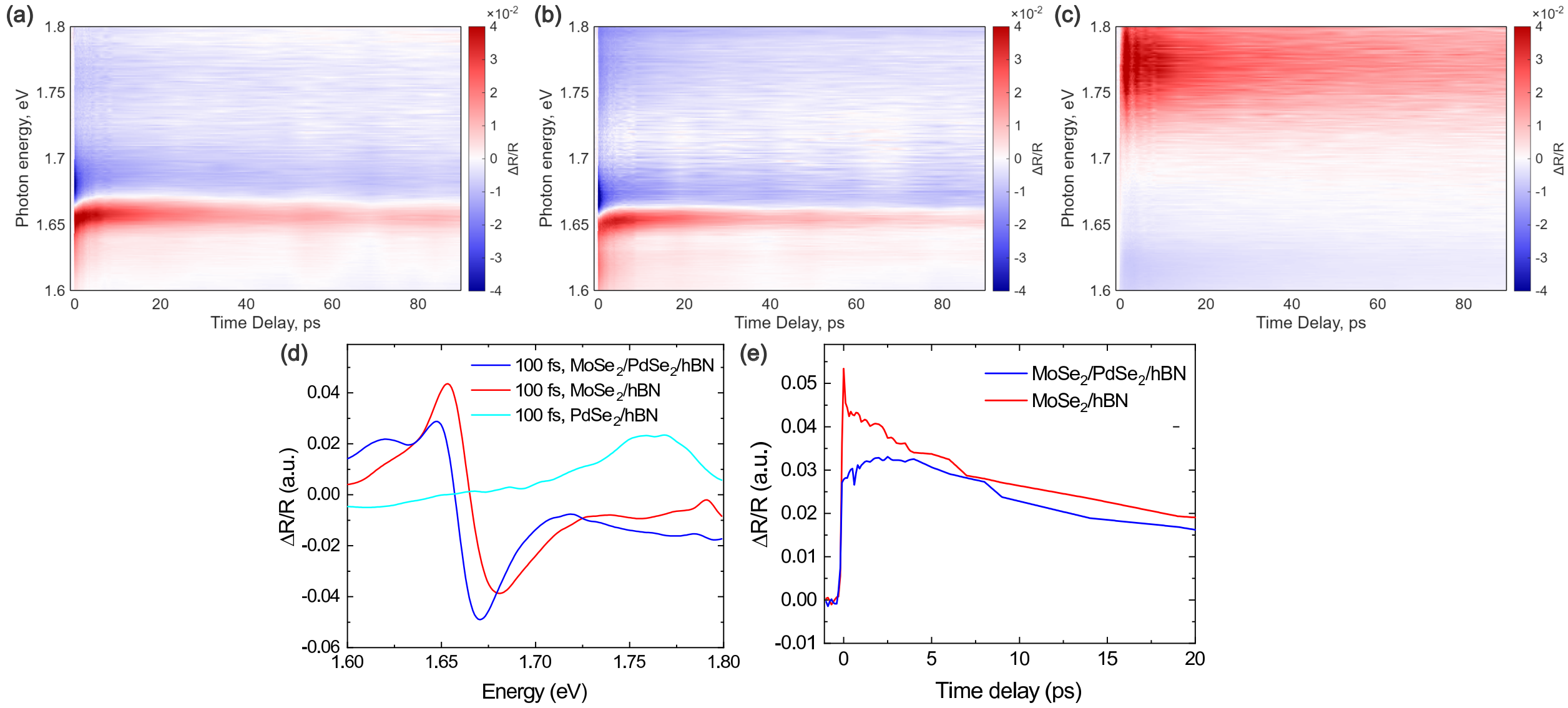

Figure S11: TR measurements of the $MoSe_2/PdSe_2$ HS and related samples at 10 K under 670 nm excitation (a) Pump-probe map of the $MoSe_2/PdSe_2$ HS (b) Pump-probe map of $MoSe_2$ (c) Pump-probe map of $PdSe_2$ (d) TR spectra of the $MoSe_2/PdSe_2$ HS and related samples (e) Time-traces extracted at the maximum of the positive peak of the A exciton in the HS (1.648 eV) and in the $MoSe_2$ sample (1.653 eV).

To directly substantiate our claim that heterojunction formation modifies exciton relaxation dynamics, we performed broadband time-resolved (TR) transient reflectivity ($\Delta R/R$) measurements over the spectral range encompassing the $MoSe_2$ A exciton. We compare the transient optical response of the $MoSe_2/PdSe_2$ HS with those of its isolated constituent layers, namely 1L $MoSe_2$ and $PdSe_2$. Further details of the experimental setup and measurement procedure can be found in Reference S21. All measurements were performed under cryogenic conditions (T = 10 K) to maximize the transient response of the $MoSe_2$ A exciton. At low temperature, the excitonic resonance becomes significantly stronger and spectrally sharper, resulting in a substantially enhanced photoinduced reflectivity signal. For all pump-probe measurements, the pump photon energy was set above the optical gaps of both materials (670 nm, 1.85 eV). Figures S11a–c show the $\Delta R/R$ maps of the HS and the two isolated constituent layers as a function of probe energy and pump-probe delay. By comparing the non-equilibrium optical response of the HS and the 1L $MoSe_2$ (Figures S11a,b), it is evident that both are dominated by the transient response of the $MoSe_2$ A exciton, centered at approximately 1.65 eV. In this spectral region, the transient response exhibits a derivative-like lineshape, arising from the combined effects of exciton bleaching due to Pauli blocking and energy renormalization associated with photoinduced changes in Coulomb screening. In contrast, the $\Delta R/R$ map of the $PdSe_2$ (Figure S11c) displays a markedly different behavior, consisting of a positive transient signal that gradually changes sign around 1.7 eV, corresponding to an interband transition of the material. Figure S11d shows the $\Delta R/R$ spectra at 100 fs, highlighting that the contribution from $PdSe_2$ is negligible in the A-exciton spectral region compared to that of the 1L $MoSe_2$. In the HS, the proximity of the $PdSe_2$ layer provides additional dielectric screening of the Coulomb interaction within the $MoSe_2$ layer, leading to a redshift of the transient bleaching signal relative to the isolated 1L.[S22] Figure S11e reports the temporal dynamics of the A-exciton resonance measured in the HS and in the isolated 1L $MoSe_2$. The two transient responses exhibit markedly different behavior within the first picosecond following photoexcitation. In the 1L $MoSe_2$, the transient dynamics are characterized by an almost instantaneous build-up (i.e., limited by the temporal resolution of the experiment) followed by two decay components within the investigated temporal window: a fast component on the order of a few hundred femtoseconds and a slower component extending over tens of picoseconds. The instantaneous build-up is attributed to the ultrafast formation of bright excitons following the photoexcitation of electron-hole pairs. The initial fast decay is assigned to intervalley scattering, which

converts optically generated bright excitons into momentum-dark excitons. The subsequent slower decay originates from a combination of processes, including exciton recombination and defect-assisted scattering. In contrast, in the HS, the A-excitonic transient signal displays an instantaneous rise followed by a second rise on a timescale of a few picoseconds. Interestingly, the relaxation dynamics do not exhibit any sub-picosecond decay component but instead show only a slow decay, similar to that observed in the isolated 1L $MoSe_2$. We tentatively attribute this distinct behavior to photoexcited carriers that are initially generated in the $PdSe_2$ layer and subsequently transferred into the $MoSe_2$ layer on a picosecond timescale, where they form excitons. Once formed, these excitons relax through the same decay channels observed in the isolated 1L, including recombination and defect-assisted scattering. The delayed rise of the A-exciton signal therefore provides indirect evidence that interlayer charge transfer from $PdSe_2$ to $MoSe_2$ competes with the intrinsic ultrafast intervalley scattering that dominates the exciton dynamics in the isolated 1L.

Transient reflectivity spectra of the HS show a delayed, picosecond-timescale rise in signal following photoexcitation, consistent with interlayer carrier transfer from $PdSe_2$ into $MoSe_2$. Given the type-I band alignment established by our DFT calculations (Figure 1b), energetic photoexcited ("hot") carriers relaxing through hybridized states may facilitate the transfer of both electrons and holes into $MoSe_2$-derived states without persistent spatial charge separation between the two layers. This is further supported by the subsequent relaxation dynamics: following the initial delayed rise, the A-exciton decay in the HS closely mirrors that of isolated 1L $MoSe_2$, showing no anomalous slowing characteristic of long-lived, spatially separated states, as has been observed in some type-II TMD-based HS.[S23] Our results are therefore consistent with the formation of co-localized intralayer excitons in $MoSe_2$, rather than long-lived spatially separated electron–hole pairs.

Temperature-dependent A- and B-exciton energies and effective spin-orbit splitting

To clarify the energetic positions of the A- and B-excitons in the HS relative to the 1L, we extracted the peak energies of both excitons as a function of temperature between 120 and 297 K from the PL spectra of the HS and 1L reference regions (Figures S12a,b). From these values, we calculated the effective spin-orbit splitting as $\Delta E_{B-A} = E_B$ - E_A for each region (Figure S12c). The A-exciton energy is only weakly perturbed in the HS, showing a nearly temperature-independent blueshift of approximately 3 meV relative to the 1L across the measured range. In contrast, the B-exciton in the HS is strongly quenched above 260 K and could therefore only be reliably resolved up to this temperature (Figure S10). Within the accessible range, ΔE_{B-A} is reduced in the HS relative to the 1L, reaching a maximum difference of about 12 meV at 220 K and decreasing toward lower temperatures, where the HS and 1L values become nearly identical. The reduced ΔE_{B-A} indicates that the B-exciton is more strongly affected by the HS than the A-exciton, consistent with stronger interlayer hybridization for the deeper B-exciton valley. The largest reduction in ΔE_{B-A} occurs in the 180–220 K range, which coincides with the temperature window where the HS PL intensity exhibits its anomalous non-monotonic behavior. These results therefore support the interpretation that interlayer coupling modifies the B-exciton more strongly than the A-exciton, while the observed quenching of B-exciton emission cannot be explained by a splitting-driven reduction in B-exciton population.

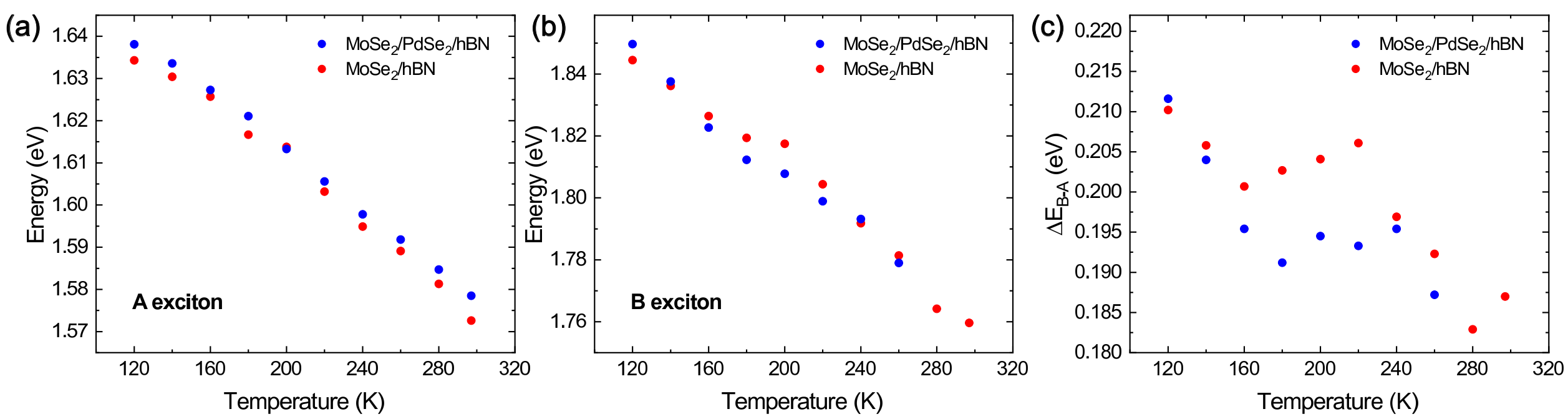

Figure S12: Temperature dependence of the a) A exciton, b) B exciton peak positions, and c) spin-orbit coupling effect in HS and 1L.

PLE spectra of MoS_2-based samples

Figure S13 presents the PLE spectra for the MoS_2-based control samples. In contrast to the broadband enhancement observed in the $MoSe_2$ system, the HS region (purple line) exhibits a systematically reduced PL intensity compared to the isolated 1L MoS_2 (yellow line) across the entire excitation range (480–625 nm), with the emission intensity lower by a factor of ∼1.8–2.5 depending on excitation wavelength.

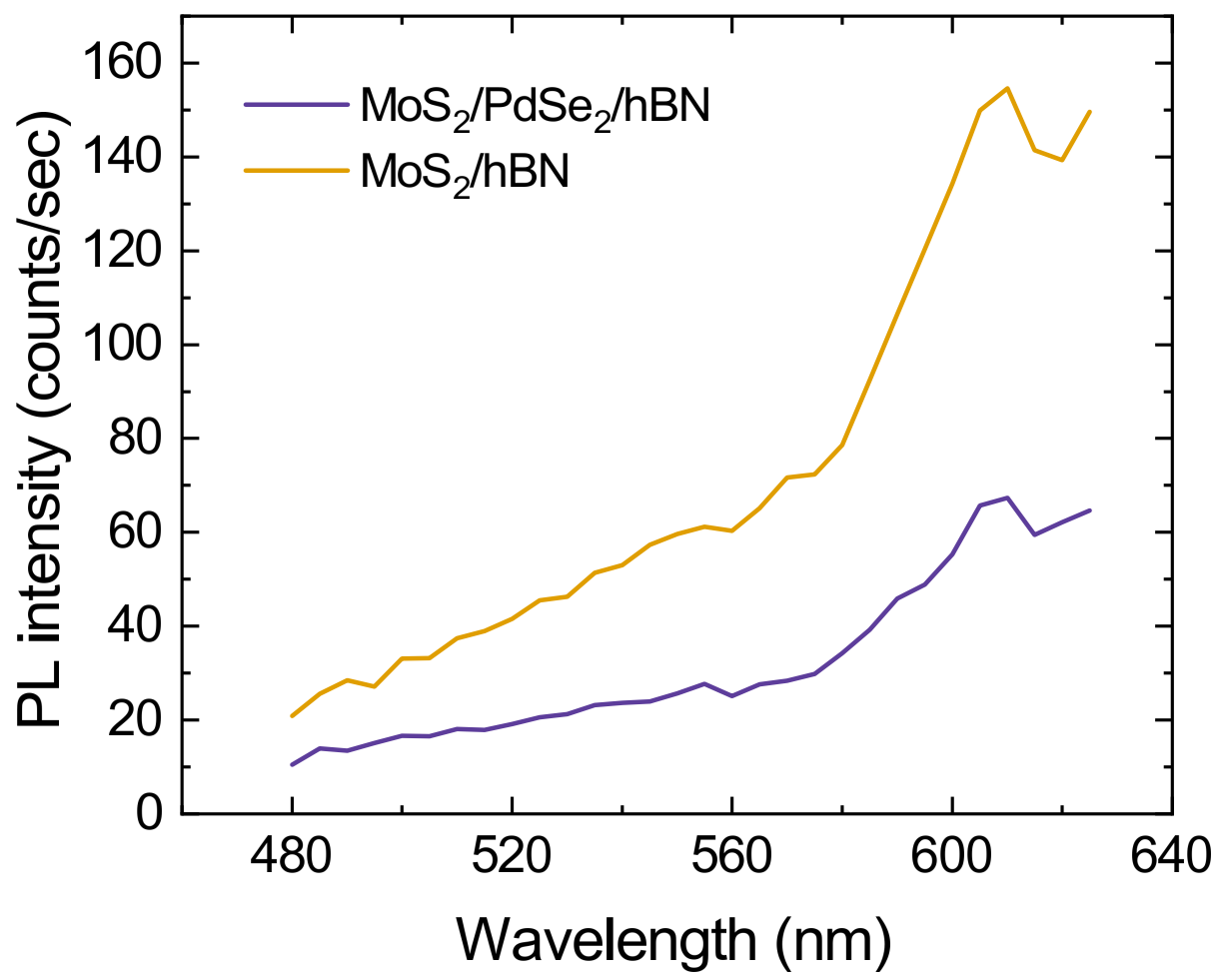

Figure S13: PLE spectra of the 1L region and the HS region

References

[S1] Castellanos-Gomez, A.; Buscema, M.; Molenaar, R.; Singh, V.; Janssen, L.; Van Der Zant, H. S. J.; Steele, G. A. Deterministic Transfer of Two-Dimensional Materials by All-Dry Viscoelastic Stamping. *2D Mater.* **2014**, *1*, 011002.

[S2] Hohenberg, P.; Kohn, W. Inhomogeneous Electron Gas. *Phys. Rev.* **1964**, *136*, B864–B871.

[S3] Kresse, G.; Furthmüller, J. Efficient Iterative Schemes for *Ab Initio* Total-Energy Calculations Using a Plane-Wave Basis Set. *Phys. Rev. B* **1996**, *54*, 11169–11186.

[S4] Kohn, W.; Sham, L. J. Self-Consistent Equations Including Exchange and Correlation Effects. *Phys. Rev.* **1965**, *140*, A1133–A1138.

[S5] Blöchl, P. E. Projector Augmented-Wave Method. *Phys. Rev. B* **1994**, *50*, 17953–17979.

[S6] Perdew, J. P.; Burke, K.; Ernzerhof, M. Generalized Gradient Approximation Made Simple. *Phys. Rev. Lett.* **1996**, *77*, 3865–3868.

[S7] Dion, M.; Rydberg, H.; Schröder, E.; Langreth, D. C.; Lundqvist, B. I. Van der Waals Density Functional for General Geometries. *Phys. Rev. Lett.* **2004**, *92*, 246401.

[S8] Oyedele, A. D. et al. $PdSe_2$: Pentagonal Two-Dimensional Layers with High Air Stability for Electronics. *J. Am. Chem. Soc.* **2017**, *139*, 14090–14097.

[S9] Hjorth Larsen, A. et al. The Atomic Simulation Environment—a Python Library for Working with Atoms. *J. Phys.: Condens. Matter* **2017**, *29*, 273002.

[S10] Ganose, A. M.; Jackson, A. J.; Scanlon, D. O. Sumo: Command-Line Tools for Plotting and Analysis of Periodic *Ab Initio* Calculations. *J. Open Source Softw.* **2018**, *3*, 717.

[S11] Puretzky, A. A.; Oyedele, A. D.; Xiao, K.; Haglund, A. V.; Sumpter, B. G.; Mandrus, D.; Geohegan, D. B.; Liang, L. Anomalous Interlayer Vibrations in Strongly Coupled Layered $PdSe_2$. *2D Mater.* **2018**, *5*, 035016.

[S12] Cowie, M.; Plougmann, R.; Benkirane, Y.; Schué, L.; Schumacher, Z.; Grütter, P. How High is a $MoSe_2$ Monolayer? *Nanotechnology* **2021**, *33*, 125706.

[S13] Pürckhauer, K.; Kirpal, D.; Weymouth, A. J.; Giessibl, F. J. Analysis of Airborne Contamination on Transition Metal Dichalcogenides with Atomic Force Microscopy Revealing That Sulfur Is the Preferred Chalcogen Atom for Devices Made in Ambient Conditions. *ACS Appl. Nano Mater.* **2019**, *2*, 2593–2598.

[S14] Alexeev, E. M.; Mullin, N.; Ares, P.; Nevison-Andrews, H.; Skrypka, O.; Godde, T.; Kozikov, A.; Hague, L.; Wang, Y.; Novoselov, K. S.; Fumagalli, L.; Hobbs, J. K.; Tartakovskii, A. I. Emergence of Highly Linearly Polarized Interlayer Exciton Emission in $MoSe_2/WSe_2$ Heterobilayers with Transfer-Induced Layer Corrugation. *ACS Nano* **2020**, *14*, 11110–11119.

[S15] Kolesnichenko, P. V.; Zhang, Q.; Yun, T.; Zheng, C.; Fuhrer, M. S.; Davis, J. A. Disentangling the effects of doping, strain and disorder in monolayer WS_2 by optical spectroscopy. *2D Materials* **2020**, *7*, 025008.

[S16] Ramzan, M. S.; Kunstmann, J.; Kuc, A. B. Tuning Valleys and Wave Functions of van der Waals Heterostructures by Varying the Number of Layers: A First-Principles Study. *Small* **2021**, *17*, 2008153.

[S17] Zhang, Y. et al. Direct Observation of the Transition from Indirect to Direct Bandgap in Atomically Thin Epitaxial $MoSe_2$. *Nat. Nanotechnol.* **2014**, *9*, 111–115.

[S18] Pakdel, S.; Rasmussen, A.; Taghizadeh, A.; Kruse, M.; Olsen, T.; Thygesen, K. S. High-throughput computational stacking reveals emergent properties in natural van der Waals bilayers. *Nat. Commun.* **2024**, *15*, 932.

[S19] Rudin, S.; Reinecke, T. L.; Segall, B. Temperature-Dependent Exciton Linewidths in Semiconductors. *Phys. Rev. B* **1990**, *42*, 11218–11231.

[S20] McCreary, K. M.; Hanbicki, A. T.; Sivaram, S. V.; Jonker, B. T. A- and B-Exciton Photoluminescence Intensity Ratio As a Measure of Sample Quality for Transition Metal Dichalcogenide Monolayers. *APL Mater.* **2018**, *6*, 111106.

[S21] Genco, A. et al. Ultrafast Dynamics of Rydberg Excitons and Their Optically Induced Charged Complexes in Encapsulated WSe_2 Monolayers. *Nano Lett.* **2025**, *25*, 7673–7681.

[S22] Chernikov, A.; Berkelbach, T. C.; Hill, H. M.; Rigosi, A.; Li, Y.; Aslan, B.; Reichman, D. R.; Hybertsen, M. S.; Heinz, T. F. Exciton Binding Energy and Nonhydrogenic Rydberg Series in Monolayer WS_2. *Phys. Rev. Lett.* **2014**, *113*, 076802.

[S23] Wang, Z.; Altmann, P.; Gadermaier, C.; Yang, Y.; Li, W.; Ghirardini, L.; Trovatello, C.; Finazzi, M.; Duò, L.; Celebrano, M.; Long, R.; Akinwande, D.; Prezhdo, O. V.; Cerullo, G.; Dal Conte, S. Phonon-Mediated Interlayer Charge Separation and Recombination in a $MoSe_2/WSe_2$ Heterostructure. *Nano Lett.* **2021**, *21*, 2165–2173.
